# Interactions between rootstocks and compost influence the active rhizosphere bacterial communities in citrus

**DOI:** 10.1186/s40168-023-01524-y

**Published:** 2023-04-20

**Authors:** Antonio Castellano-Hinojosa, Ute Albrecht, Sarah L. Strauss

**Affiliations:** 1 Present Address: Department of Soil, Water, and Ecosystem Sciences, Southwest Florida Research and Education Center, Institute of Food and Agricultural Sciences, University of Florida, 2685 State Rd 29N, Immokalee, FL 34142 USA; 2 Department of Horticultural Sciences, Southwest Florida Research and Education Center, Institute of Food and Agricultural Sciences, University of Florida, 2685 State Rd 29N, Immokalee, FL 34142 USA

**Keywords:** Active rhizobiome, Rhizosphere, Tree crops, RNA, Citrus, Rootstock selection, Bacterial recruitment, Multinutrient cycling, Predicted functions

## Abstract

**Background:**

While the rootstock genotype (belowground part of a plant) can impact rhizosphere microbial communities, few studies have examined the relationships between rootstock genotype-based recruitment of active rhizosphere bacterial communities and the availability of root nutrients for plant uptake. Rootstocks are developed to provide resistance to disease or tolerance of abiotic stresses, and compost application is a common practice to also control biotic and abiotic stresses in crops. In this field study, we examined: (i) the effect of four citrus rootstocks and/or compost application on the abundance, diversity, composition, and predicted functionality of active rhizosphere bacterial communities, and (ii) the relationships between active rhizosphere bacterial communities and root nutrient concentrations, with identification of bacterial taxa significantly correlated with changes in root nutrients in the rhizosphere.

**Results:**

The rootstock genotype determined differences in the diversity of active rhizosphere bacterial communities and also impacted how compost altered the abundance, diversity, composition, and predicted functions of these active communities. Variations in the active bacterial rhizobiome were strongly linked to root nutrient cycling, and these interactions were root-nutrient- and rootstock-specific. Direct positive relationships between enriched taxa in treated soils and specific root nutrients were detected, and potentially important taxa for root nutrient uptake were identified. Significant differences in specific predicted functions were related to soil nutrient cycling (carbon, nitrogen, and tryptophan metabolisms) in the active bacterial rhizobiome among rootstocks, particularly in soils treated with compost.

**Conclusions:**

This study illustrates that interactions between citrus rootstocks and compost can influence active rhizosphere bacterial communities, which impact root nutrient concentrations. In particular, the response of the rhizobiome bacterial abundance, diversity, and community composition to compost was determined by the rootstock. Specific bacterial taxa therefore appear to be driving changes in root nutrient concentrations in the active rhizobiome of different citrus rootstocks. Several potential functions of active bacterial rhizobiomes recruited by different citrus rootstocks did not appear to be redundant but rather rootstock-specific. Together, these findings have important agronomic implications as they indicate the potential for agricultural production systems to maximize benefits from rhizobiomes through the choice of selected rootstocks and the application of compost.

Video Abstract

**Supplementary Information:**

The online version contains supplementary material available at 10.1186/s40168-023-01524-y.

## Background

The rhizosphere is the region around the root characterized by high concentrations of plant-derived organic exudates that serve as signal molecules and nutrient sources for microbial recruitment [1, 2]. The microbial communities of the rhizosphere, which constitute the “rhizobiome,” are essential for plant health as they can increase plant nutrient uptake and resistance to several biotic and abiotic stresses through mechanisms including induced systemic resistance, suppression of plant pathogens, and solubilization of soil minerals [3–6].

Most fruit tree crops are composed of two parts: the aboveground fruit-bearing part, the scion, and the belowground part, the rootstock, which provides anchorage and is responsible for water and nutrient uptake. The scion and rootstock, which are often genetically different, are joined through the process of grafting [7, 8]. New rootstocks are developed to adapt to soilborne stresses and diseases and to modulate the horticultural characteristics of the scion. The history of rootstock use and breeding in modern citrus production has been shaped by diseases such as *Phytophthora* root rot, *Citrus tristeza virus*, and more recently huanglongbing (HLB, a.k.a. citrus greening) [9]. The rootstock genotype cannot only modulate horticultural traits such as tree size and productivity [10] but can also influence the composition of the rhizosphere microbial communities [2]. The genotype influence on the rhizobiome can even extend to within-species differences as demonstrated in grapes [11–14], apples [15–17], tomatoes [18], and *Populus* sp. [19, 20].

Root health is a critical factor for tree growth as it directly influences a tree’s ability to cope with adverse biotic and abiotic stressors. Despite the importance of the rhizobiome for plant nutrient availability [21], few studies have examined the direct link between the rootstock genotype-based recruitment of rhizosphere bacterial communities and the availability of root nutrients for plant uptake. The potential impacts of plant genotype on the rhizobiome composition and nutrient availability are particularly relevant because they suggest the potential for agricultural production systems to maximize benefits from rhizobiomes indirectly through the choice of rootstocks. Just as rootstocks are bred to resist specific soilborne diseases, plant genotypes with desired phenotypes can be used as a microbiome engineering tool to select candidate taxa (e.g., to serve as biofertilizers or biocontrol agents) for agricultural microbiome engineering [22–24]. In addition, the study of the host genes associated with the selection of microbial communities can be used to support microbiome-focused crop breeding [25].

Citrus is a globally important perennial fruit crop, but its production faces challenges, particularly from the devastating disease HLB [26–28]. Several strategies, including the use of selected rootstock genotypes [9, 29], ground application of specific nutrients [30], and soil amendments (e.g., compost and plant biostimulants such as humic substances, seaweed extracts, and microbial inoculants) [31, 32], have been proposed to improve root health and crop production in citrus. In addition, there is increased interest to understand the composition and function of the citrus microbiome to help optimize and maximize future agricultural microbiome engineering solutions [33–35]. In citrus, rootstock selection is essential for the success or failure of a citrus operation [36], and the benefits of using specially selected rootstocks has been documented in numerous publications [37–42]. Recent studies have also shown that the root metabolic composition may differ among citrus rootstocks [43–45]. This raises the question of whether different citrus rootstocks may recruit distinct rhizosphere bacterial communities that could impact root nutrient cycling.

Florida is one of the largest citrus producers in the USA with more than 60 million trees on 143,000 harvested ha [46]. Most citrus in Florida is grown on naturally infertile soils that have little organic matter and are unable to retain more than a minimal amount of soluble nutrients [47], directly affecting the establishment of trees during the early phase when rapid development of the tree canopy is critical. This situation is exacerbated when trees become infected with HLB and fibrous roots start to decline [48]. Increasing soil carbon availability through the application of compost can provide a wide range of benefits for root health and production, including improving nutrient and water retention and nutrient availability [49, 50]. Application of compost can also impact the soil microbiome and increase microbial diversity [51, 52], which has been linked to reduced disease incidence [53, 54]. A recent study showed that compost application increased the bacterial diversity in the apple rhizosphere of two rootstocks, and that interactions between compost and rootstocks controlled variations in the rhizobiome composition that may determine increases in tree biomass [17]. However, the interaction between compost and rootstocks in the citrus rhizobiome has not been explored, nor has the relationship between rhizobiome taxa and root nutrient concentrations. A recent work showed that predicted bacterial functions in the rhizobiome of grapes were similar among different rootstocks [12]. This suggests that the potential functions of bacterial rhizobiomes recruited by different rootstocks of the same crop may be redundant and evenly spread. Whether this is the case for other crops such as citrus remains to be determined, as well as how the application of compost may impact microbial functions in the citrus rhizobiome and root nutrient availability.

To date, the study of rootstock effects on the rhizobiome of crops has been predominately performed using a DNA-based amplicon sequencing approach. However, RNA-based estimates can be more accurate for soil microbiome studies [55–58] since relic DNA is abundant in soil and obscures estimates of soil microbial diversity. In addition, highly active microbial taxa may be rare or even absent from DNA-based approaches for the study of soil microbial communities [55–58]. Therefore, we used extracted 16S rRNA from the citrus rhizosphere to: (1) examine the effect of different citrus rootstocks and/or compost on the abundance, diversity, composition, and predicted functionality of active rhizosphere bacterial communities, and (2) determine the relationships between active rhizosphere bacterial communities and root nutrient concentrations and identify potential bacterial taxa correlated with changes in root nutrients. We hypothesized that the rootstock genotype determines variations in diversity and composition of the rhizobiome, and that the rhizobiome bacterial community is richer and more diverse in soils treated with compost compared to the control, resulting in greater root nutrient concentrations.

## Methods

### Study site, experimental design, and management

The field study was carried out in a commercial citrus orchard in Southwest Florida (Hendry County, FL, USA) under HLB-endemic conditions [28]. The soil at the study site is a sandy spodosol according to the soil taxonomy of USDA [59], consisting of a surface layer, which is low in organic matter (< 1.5%) and soil N content [< 10 mg/kg of ammonium (NH_4_^+^) + nitrate (NO_3_^−^)], and a subsurface layer with poor drainage [60]. Trees were planted in August 2019 in double rows on raised beds separated by furrows at a spacing of 3.7 m within rows and 7.6 m between rows (358 trees/ha). General management of the orchard followed practices determined by the orchard operator and included seepage irrigation, insecticide, herbicide and fertilizer applications, and other standard management practices. Trees consisted of ‘Valencia’ sweet orange scion (*Citrus sinensis*) on four different rootstocks: (i) X-639 (*C. reticulata* ‘Cleopatra’ × *Poncirus trifoliata* ‘Rubidoux’); (ii) US-802 (*C. maxima* ‘Siamese’ × *P. trifoliata* ‘Gotha Road’); (iii) US-812 (*C. reticulata* ‘Sunki’ × *P. trifoliata* ‘Benecke’); and (iv) US-897 (*C. reticulata* ‘Cleopatra’ × *P. trifoliata* ‘Flying Dragon’). Two treatments were assayed: compost and no compost (control).

The field experiment was a randomized split-plot design with treatment (compost or control) as the main plot and rootstock (X-639, US-802, US-812, or US-897) as the subplot (Supplementary Fig. S1). Plots were arranged in eight blocks (16 beds) across a 9-ha experimental site with each block containing two beds either treated with compost or untreated (control). Each bed contained 200 experimental trees, 100 per row, arranged in sets of 50 trees on each of the four rootstocks (Supplementary Fig. S1). Subplots consisted of one bed containing compost and one bed without compost. There were 64 experimental units in total (8 blocks × 2 treatments × 4 rootstocks). Two months after planting (November), compost was applied at a rate of 12.4 tons/ha and incorporated in beds by a shallow till; the other half of the beds did not receive any compost. Following this initial application, compost was applied every 6 months at the same rate (12.4 tons/ha) by broadcast spreading. The locally sourced compost (Kastco Agriculture Service, Naples, FL, USA) was made from yard waste. The physicochemical characteristics of the compost were as follows: C:N ratio, 24.9; organic matter, 23.6%; pH in water, 7.7; total solids, 51.14%; conductivity, 3.1 mS/cm; phosphorus (P), 0.08%; potassium (K), 0.26%; sulfur (S), 0.09%; calcium (Ca), 3.28%; magnesium (Mg), 0.31%; iron (Fe), 2500 ppm; manganese (Mn), 67.5 ppm; and boron (B), 100 ppm.

### Rhizosphere sample collection

Fibrous roots (≤ 1 mm in diameter) with soil attached were collected in August 2021, two years after planting and after 4 consecutive compost applications, from eight trees from each experimental unit under the canopy, and pooled. Roots were separated in the field and used for the following: (1) root nutrient analysis (about 50 g of roots) and (2) isolation of rhizosphere soil and subsequent RNA extraction (about 10 g of roots). Fibrous roots for microbial analyses were placed in 50-mL sterile centrifuge tubes, immediately flash frozen in liquid nitrogen, and stored at −80° until analysis. Rhizosphere soil for RNA extraction was isolated using sterile phosphate-buffered saline (PBS) solution as described previously [32].

### Root nutrient analysis

Root samples for quantification of macro (N, P, K, Mg, Ca, and S) and micronutrients (B, Zn, Mn, Fe, and Cu) were sent to a commercial laboratory (Waters Agricultural Laboratories Inc., Camilla, GA, USA) and analyzed using inductively coupled plasma (ICP) emission spectroscopy [61].

### RNA extraction and reverse transcription of RNA to cDNA

RNA from 1 g of rhizosphere soil was extracted using the RNA PowerSoil^®^ Total RNA Isolation kit (Qiagen, USA) according to manufacturer’s instructions. The RNA obtained was quantified using the Qubit^™^ RNA High Sensitivity assay kit (Thermo Scientific, USA), treated with DNase I (RNase free) (Qiagen, USA) to remove co-extracted DNA following the manufacturer’s directions, and kept at −80 °C until analysis. The High-Capacity cDNA Reverse Transcription Kit was used for reverse transcription reactions with RNase inhibitor (Thermo Scientific, USA), following the manufacturer’s instructions, and using 150–200 ng RNA in a final volume of 20 μL. Synthesis of cDNA was achieved with the use of random primers. The concentration of cDNA was measured using the Qubit™ DNA High Sensitivity assay kit (Thermo Scientific, USA) and kept at −80 °C until analysis.

### qPCR assays

The total abundance of active bacterial communities was determined by quantitative PCR (qPCR) using the 16S rRNA gene as a molecular marker and cDNA as a template. Quantitative amplifications were performed following the procedures, primers, and thermal conditions previously described by Castellano-Hinojosa et al. [62] and using a QuantStudio 3 Real-Time PCR system (ThermoFisher, USA). Calibration curves had a correlation coefficient *r*^2^ > 0.99 in all assays. The efficiency of PCR amplification was between 90 and 100%.

### Library preparation and sequencing analysis

The extracted cDNA was sent for sequencing at the DNA Services Facility at the University of Illinois, Chicago, IL, USA. The V4 region of the bacterial 16S rRNA gene was amplified using the 515Fa and 926R primers following the Earth Microbiome Project protocol [63]. Raw reads were analyzed using QIIME2 v2018.4 following the procedures described in full detail in Castellano-Hinojosa and Strauss [64]. Briefly, bacterial rRNA gene sequence reads were assembled and dereplicated using DADA2 [65] with the paired-end setting into representative amplicon sequence variants (ASVs). ASVs were assigned to the SILVA 132 database [66] using the naïve Bayes classifier in QIIME2 [67]. After quality filtering, denoising, and chimera removal, 4743365 16S rRNA sequences (mean of 74115 per sample) were obtained from the total of 64 samples. Rarefaction curves reached saturation for all samples, indicating sequencing depth was sufficient (data not shown). Raw sequence data were deposited in NCBI’s Sequence Read Archive under BioProject PRJNA837574.

### Analysis of the diversity and composition of active rhizosphere bacterial communities

Alpha (Shannon and Inverse Simpson) and beta-diversity analyses were performed on log-normalized data to avoid rarefaction errors using the R package “phyloseq” v1.24.0 [68, 69]. Beta-diversity analysis included a nonmetric multidimensional scaling (NMDS) on Bray-Curtis distance. Differences in community composition between rootstocks, treatments, and their interaction were tested by permutational analysis of variance (PERMANOVA). The nonparametric analysis ANOSIM based on the relative abundance of the bacterial ASVs was used to examine similarities between rootstocks for each treatment. R values close to 1 indicate dissimilarity between treatments. Differentially abundant bacterial taxa between treatments at the phylum and genus taxonomic levels were detected using the DESeq2 package [70]. *p*-values ≤ 0.05 were considered significant.

### Functional characteristics of active rhizosphere bacterial communities

PICRUSt2 was used to predict the functional capabilities at the category and pathway levels of active rhizosphere bacterial communities based on 16S rRNA gene amplicon data as described by Douglas et al. [71]. Significant differences in functional characteristics between groups of samples were studied using the Welch’s *t*-test, followed by Benjamini–Hochberg-FDR as a multiple test correction [72].

### Quantification of a root multinutrient cycling index

Belowground soil biodiversity has a key role in determining ecosystem functioning [73]. Because bacterial communities perform multiple simultaneous functions (multifunctionality), rather than a single measurable process, we constructed a root multinutrient cycling index (MNC) analogous to the widely used multifunctionality index [74–78] using the root nutrients N, P, K, Mg, Ca, S, B, Zn, Mn, Fe, and Cu. These nutrients deliver some of the fundamental supporting and regulating ecosystem services [74–77] and are essential for crop growth, particularly for citrus trees in HLB-endemic conditions [79]. For example, two of the most limiting nutrients for primary production in terrestrial ecosystems are N and P [80]. Potassium, the third essential macronutrient for plants, is involved in numerous biological processes that contribute to crop growth, including protein synthesis, enzyme activation, and photosynthesis [79]. Calcium (Ca) plays a role in cell division and elongation [81]. Magnesium is essential for chlorophyll and an important cofactor of several enzymes [79]. Sulfur acts as a signaling molecule in stress management as well as normal metabolic processes [82]. Micronutrients such as B, Zn, Mn, Fe, and Cu are essential to achieve high plant productivity [79]. Each of the eleven root nutrients were normalized (log-transformed) and standardized using the Z-score transformation. To derive a quantitative MNC value for each treatment and rootstock, we averaged the standardized scores of all individual nutrient variables [74, 76]. The MNC index provides a straightforward and interpretable measure of the ability of bacterial communities to sustain multiple functions simultaneously [74–78]. It measures all functions on a common scale of standard deviation units, has good statistical properties, and shows good correlating with previously established indices that quantify multifunctionality [83]. Pearson’s correlation analysis was used to estimate the relationship between bacterial abundance, alpha- and beta-diversity, and MNC using the cor.test function in R.

### Identification of the active taxonomic and predicted functional core rhizobiome

We studied whether the application of compost impacts the active taxonomic and predicted functional core rhizobiome of citrus. ASVs (at the genus level) and Kyoto Encyclopedia of Genes and Genomes (KEGG) pathways [84] present in at least 75% of the samples were identified as the taxonomic and predicted functional core rhizobiome, respectively, in the control and treated soils [64, 85].

### Statistical analyses

All statistical analyses were conducted in the R environment (v3.5.1; http://www.r-project.org/). Means of bacterial abundance, alpha diversity, and root nutrients were compared via linear mixed-effects (LME) models, with rootstock (X-639, US-802, US-812, or US-897) and treatment (compost or control) considered random factors and dependent variables, respectively, by using the function “lme” in the “nlme” package. Significant effects were determined by analysis of variance (ANOVA) (*p* ≤ 0.05). A Tukey’s post hoc test was calculated by using the function “lsmeans.” We used a multiple regression model with variance decomposition analysis to evaluate the relative importance of the differentially abundant taxa between treatments for explaining variations in root nutrients using the R package “relaimpo” [86]. Structural equation modelling (SEM) [87] was used to evaluate the relationships between rootstock, compost, MNC, bacterial abundance, alpha- and beta-diversity, and predicted functionality. The a priori model is shown in Supplementary Fig. S2. Path coefficients of the model and their associated *p*-values were calculated [87]. We used bootstrap to test the probability that a path coefficient differs from zero since some of the variables introduced were not normally distributed [88]. When these data manipulations were completed, we parameterized our model using our data set and tested its overall goodness of fit. We used the χ^2^ test (*χ*^2^; the model has a good fit when *χ*^2^ is ≤ 2 and *P* is ≥ 0.05) [89] and the root mean square error (MSE) of approximation (RMSEA; the model has a good fit when RMSEA is ∼≤ 0.05 and *P* is ∼≥ 0.05) [89]. All SEM analyses were conducted using AMOS 20.0 (AMOS IBM, USA). Significant differences in the relative abundance of ASVs and pathways between taxonomic and predicted functional core rhizobiomes in the control vs. treated soils were calculated using the Welch’s *t*-test [90] and the Benjamini–Hochberg False Discovery Rate (FDR) multiple-test correction [72] using the R package “sgof.”

## Results

### Root nutrient analysis

Treatment with compost had a significant effect on root K, Mg, and Mn concentrations (Supplementary Fig. S3). The K concentration was significantly lower in roots from US-897 in treated soils compared to the control. Significantly greater Mg concentrations were detected in roots from US-802 and US-812 in treated soils compared to the controls. For US-802, US-812, and US-897, the Mn concentrations were also significantly greater in roots from the treated soils compared to the controls.

Rootstock had a significant effect on Ca, S, and Mn concentrations (Supplementary Fig. S3). In control soils, roots from US-802 had significantly higher Ca concentrations compared to US-812. In soils treated with compost, significantly higher Ca concentrations were detected in roots from US-802 compared to X-639. The S concentrations were also significantly higher in roots from US-802 and US-812 compared to X-639. Significantly higher Mn concentrations were detected in roots from US-812 and US-897 compared to US-802. There were no significant differences in N, P, S, B, Zn, Fe, and Cu concentrations among rootstocks and treatments (Supplementary Fig. S3).

### Abundance and alpha- and beta-diversity of active rhizosphere bacterial communities

Rootstock, treatment, and rootstock and treatment interaction had a significant effect on the abundance of rhizosphere bacteria (Supplementary Fig. S4). A significantly greater number of bacteria were detected in the rhizobiome of US-812 and US-897 compared to US-802 and X-639 in treated soils, whereas there were no differences in bacterial abundance between rootstocks in the control soils (Supplementary Fig. S4).

**Fig. 1:**
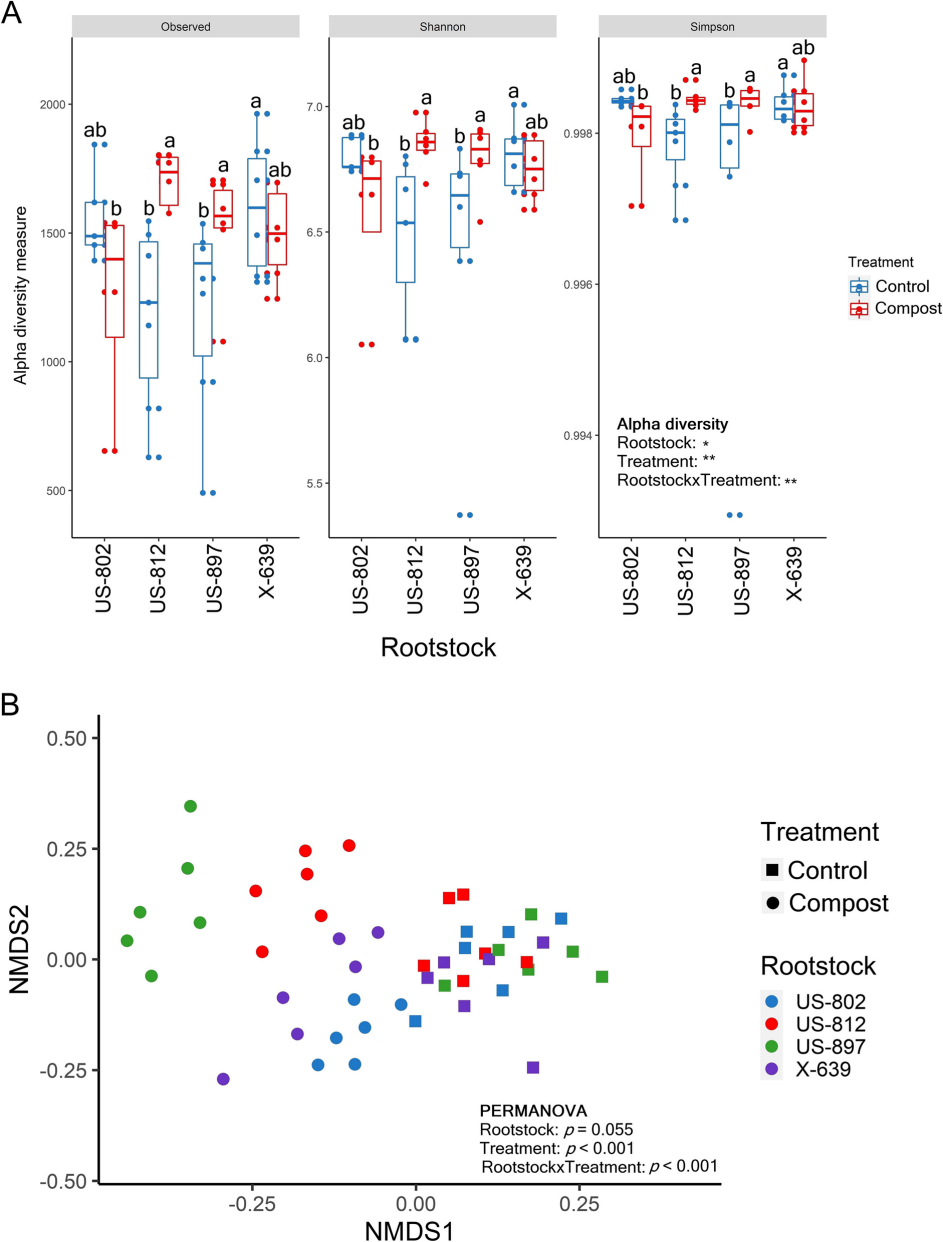
**A** Number of observed ASVs and values of Shannon and inverse Simpson diversity indices of active bacterial communities in the rhizosphere of citrus trees on four different rootstocks. Soils were untreated (control) or treated with compost. Different letters above the bars indicate significant differences between rootstocks and treatments (linear mixed-effect model and Tukey’s HSD; *n* = 8; *p ≤ 0.05; **p ≤ 0.01; ***p ≤ 0.001). Values are expressed as mean with standard error. **B** Nonmetric multidimensional scaling (NMDS) plot on Bray-Curtis distance for active bacterial communities in the rhizosphere of four different rootstocks. Differences in community composition between rootstocks, treatments, and their interactions were tested by PERMANOVA. Stress = 0.124

Alpha-diversity was significantly affected by rootstock, treatment, and rootstock and treatment interaction (Fig. 1). Compost application significantly increased the number of observed ASVs and the values of the Shannon and Simpson indices for US-812 and US-897 compared to the control soils (Fig. 1A). In the control soils, alpha-diversity was significantly greater in the rhizobiome of X-639 compared to US-812 and US-897 (Fig. 1A). In the treated soils, US-802 had significantly lower alpha-diversity compared to US-812 and US-897 (Fig. 1A).

NMDS analysis on Bray-Curtis distance together with a PERMANOVA analysis showed significant differences in the composition of bacterial communities between treatments and rootstock and treatment interaction (*p* < 0.001) and no significant differences between rootstocks (*p* = 0.055) (Fig. 1B). A subsequent ANOSIM analysis showed there were no significant differences in beta-diversity between rootstocks for the control soils, but that the composition of the bacterial community significantly differed between rootstocks in the treated soil except for US-812 vs. X-639 and US-897 vs. X-639 (Supplementary Table S2).

### Bacterial community composition and differentially abundant taxa between rootstocks and treatments

**Fig. 2:**
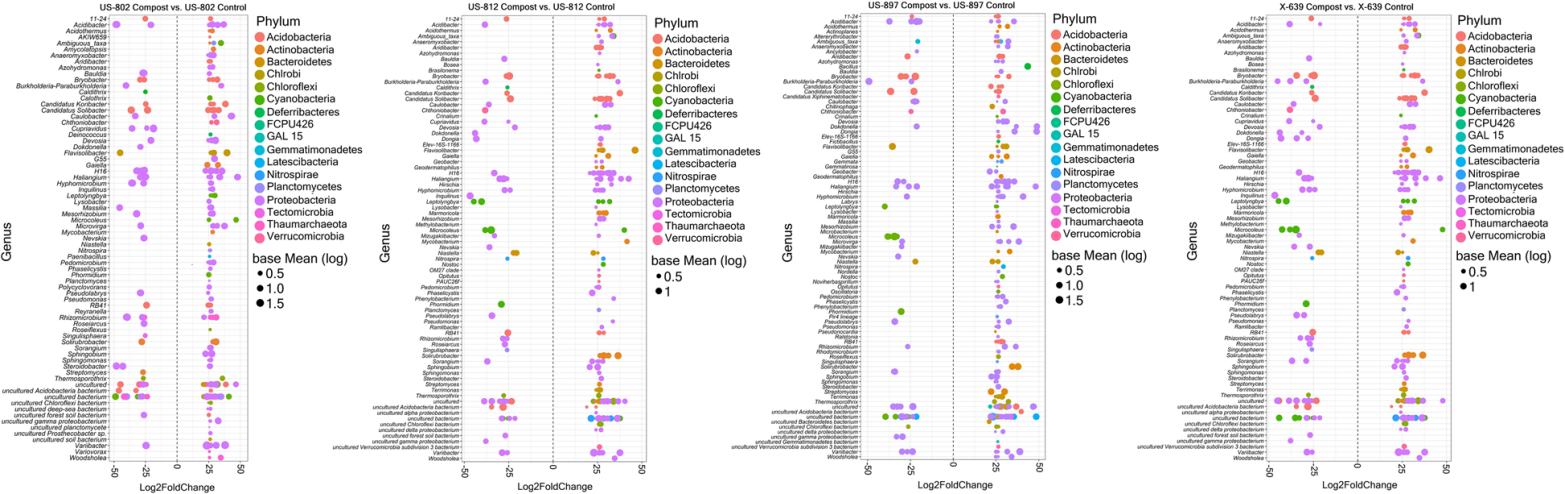
Differentially abundant ASVs at the genus taxonomic level between compost and control treatments for each rootstock. The fold change is shown on the *X*-axis, and genera are listed on the *Y*-axis. Each colored dot represents an ASV that was identified by DESeq2 analysis as significantly differentially abundant (*p* ≤ 0.05)

On average, Proteobacteria (48.25%), Acidobacteria (12.9%), Chloroflexi (8.5%), Cyanobacteria (6.4%), *Bacteroidetes* (6.1%), Actinobacteria (5.9%), and Planctomycetes (5.8%) were the most abundant bacterial phyla across all rootstocks and treatments (Supplementary Fig. S5). Active bacterial ASVs significantly enriched and depleted between treatments for each of the rootstocks were identified at the phylum (Supplementary Fig. S6) and genus (Fig. 2) taxonomic levels. Regardless of the rootstock, compost application significantly increased the relative abundance of ASVs belonging to the phyla Firmicutes, Latescibacteria, Tectomicrobia, and candidate phyla GAL15 and FCPU426 compared to control soils (Supplementary Fig. S6). However, more abundant phyla such as Proteobacteria, Nitrospirae, Cyanobacteria, Chloroflexi, *Bacteroidetes*, Actinobacteria, and Acidobacteria had both enriched and depleted taxa within the same phyla in soils treated with compost compared to the controls, suggesting treatment effects on bacterial taxa assigned to these phyla were not phylum-specific (Supplementary Fig. S6).

Significantly enriched (e.g., *Acidothermus*, *Anaeromyxobacter*, *Aridibacter*, *Azohydromonas*, *Crinalium*, *Lysobacter*, *Pseudomonas*, *Nitrospira*, *Sphingobium*, *Sphingomonas*, *Planctomyces*, *Pedomicrobium*, and *Woodsholea*) and depleted genera (e.g., *Caldithrix*, *Cupriavidus*, and *Nevskia*) in the treated soils were identified across all rootstocks compared to the control soils (Fig. 2). Other genera had both significantly enriched and depleted ASVs such as *Acidibacter*, *Bauldia*, *Bryobacter*, *Burkholderia*, *Devosia*, *Hyphomicrobium*, *Mesorhizobium*, *Microvirga*, *Varibacter*, and *Rhizomicrobium*. Overall, US-812 and US-897 showed a greater proportion of enriched rather than depleted (78% and 22% and 75% and 25%, respectively) ASVs compared to US-802 (60% and 40%, respectively) and X-639 (62% and 38%, respectively).

### Potential contributions of differentially abundant active taxa to root nutrient concentrations

**Fig. 3:**
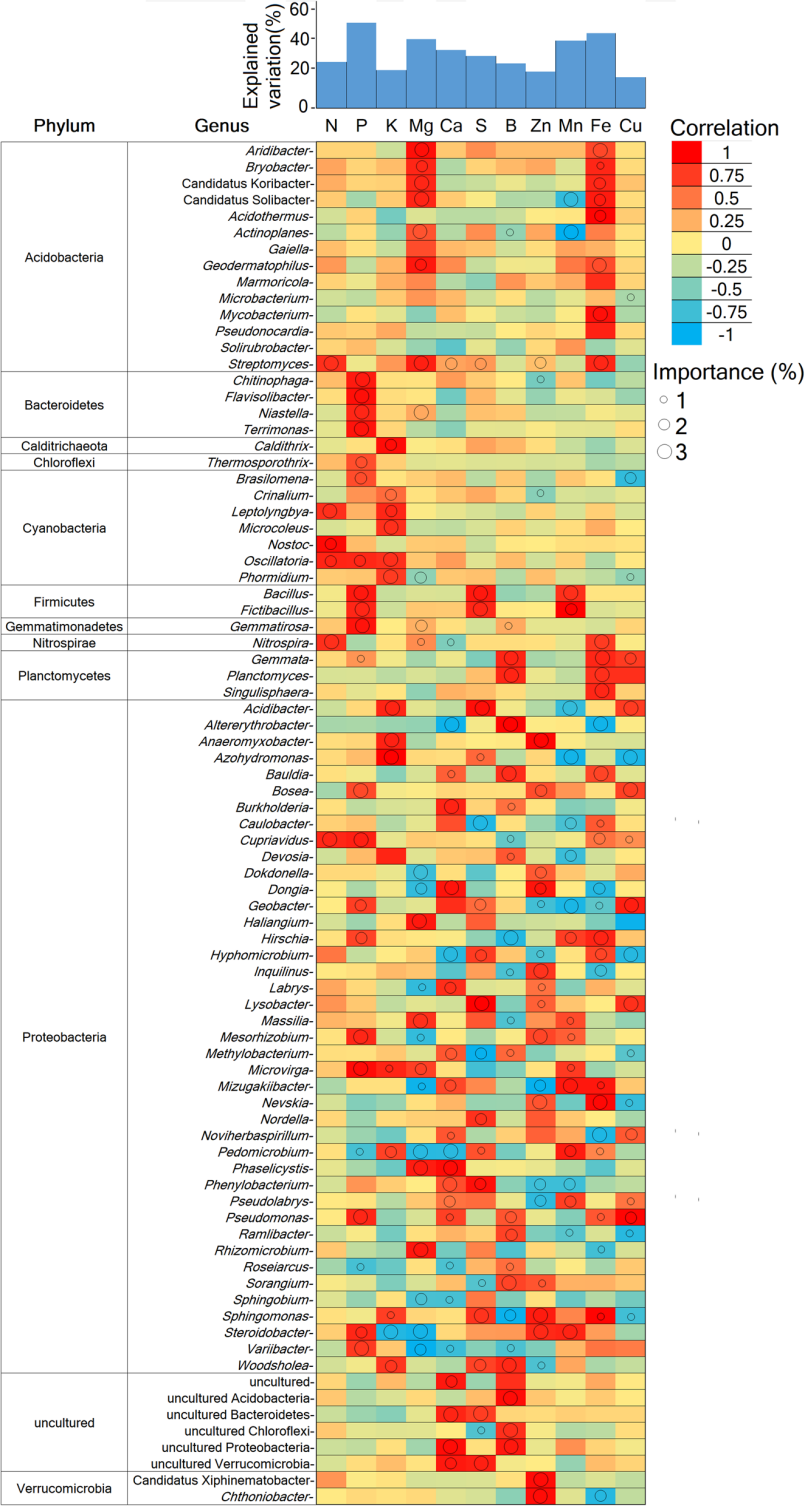
Correlations between the relative abundance of differentially abundant taxa and root nutrients. Circle size represents the variable’s importance (i.e., proportion of explained variation calculated via multiple regression modeling and variance decomposition analysis). The shading from blue to red represents low- to high-positive Spearman correlation coefficients

All differently abundant active bacterial genera contributed to the variations in root nutrient concentrations (Fig. 3). For example, genera belonging to Acidobacteria such as *Aridibacter*, *Bryobacter*, *Candidatus Koribacter*, and *Candidatus* Solibacter were found important and positively correlated with root Mg and Fe concentrations, whereas others such as *Streptomyces* were important for predicting changes in root N, Mg, Ca, S, Zn, and Fe concentrations. Genera assigned to *Bacteriodetes*, such as *Chitinophaga*, *Flavisolibacter*, *Niastella*, and *Terrimonas*, were important and positively correlated with root P concentrations. *Callithrix* (Calditrichaeota phylum) and *Thermosporothrix* (Chloroflexi) were positively correlated with root K and P, respectively (Fig. 3). Genera belonging to Cyanobacteria, such as *Leptolyngbya*, *Nostoc*, *Oscillatoria*, and *Microcoleus*, were important for predicting changes in root N, P, and K concentrations and were positively correlated with these root nutrients. *Bacillus* and *Fictibacillus* (Firmicutes phylum) were positively correlated with root P, S, and Mn, whereas *Nitrospira* (Nitrospirae phylum) was important for predicting changes in root N and Fe (Fig. 3). Genera belonging to Planctomycetes, such as *Gemmata* and *Planctomyces*, were important and positively correlated with Fe and Cu, whereas those assigned to Verrucomicrobia phylum (e.g., *Candidatus* Xiphinematobacter and *Chthoniobacter*) were positively correlated with Zn. Within Proteobacteria, there were 41 genera that were important and positively or negatively correlated with all root nutrients (Fig. 3). These included *Burkholderia*, *Dongia*, and *Methylobacterium* which were positively correlated with root Ca and *Hyphomicrobium* and *Pedomicrobium* which were negatively correlated with this root nutrient (Fig. 3).

### Relationships between microbial diversity and the MNC

**Fig. 4:**
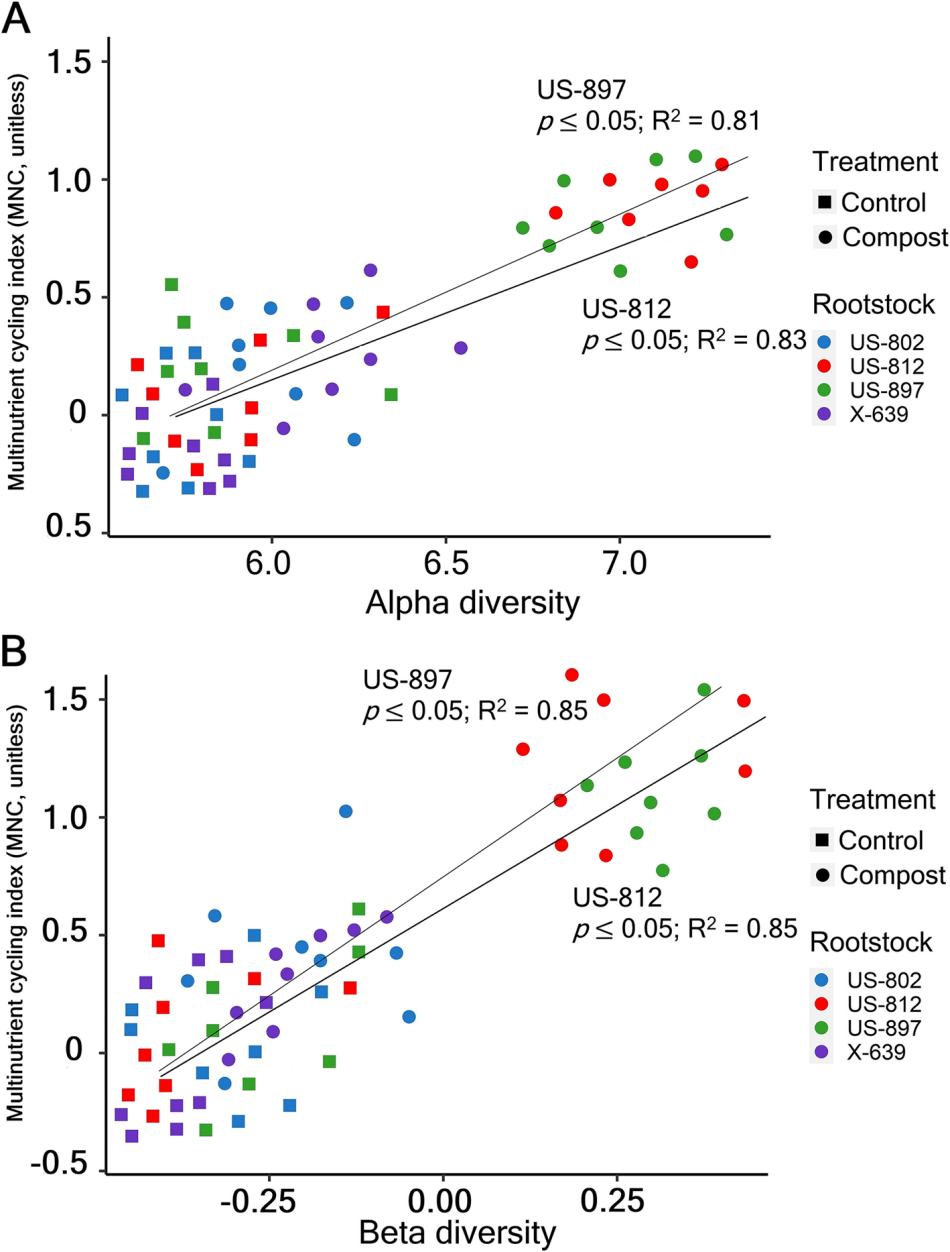
Relationships between bacterial alpha **A** and beta **B** diversity and the multinutrient cycling index (MNC) for each rootstock and treatment. The bacterial *α*-diversity was calculated as the average value of the Shannon index after minimum-maximum normalization. The microbial community composition (*β*-diversity) was estimated using the first axis of the nonmetric multidimensional scaling (NMDS) analysis. Solid lines denote significant Pearson correlations (*p* ≤ 0.05)

The MNC index increased in soils treated with compost compared to the controls for US-812 and US-897 (Fig. 4). There were significant positive relationships between bacterial alpha- and beta-diversity and MNC for US-812 and US-897 rootstocks (Fig. 4). Concerning each component of the multinutrient cycling index, alpha- and beta-diversity significantly and positively correlated with root Mg and Mn concentrations for all rootstocks and with root Zn for US-812 and US-897 (Supplementary Fig. S7 A, B). Root K was significantly and negatively correlated with alpha- and beta-diversity for US-812 and US-897 (Supplementary Fig. S7 A, B). Root N, P, and Ca concentrations were significantly and positively correlated alpha- and beta-diversity for US-812. Root Cu was positively correlated with beta-diversity for all rootstocks (Supplementary Fig. S7B).

### Predicted functional traits of active rhizosphere bacterial communities

**Fig. 5:**
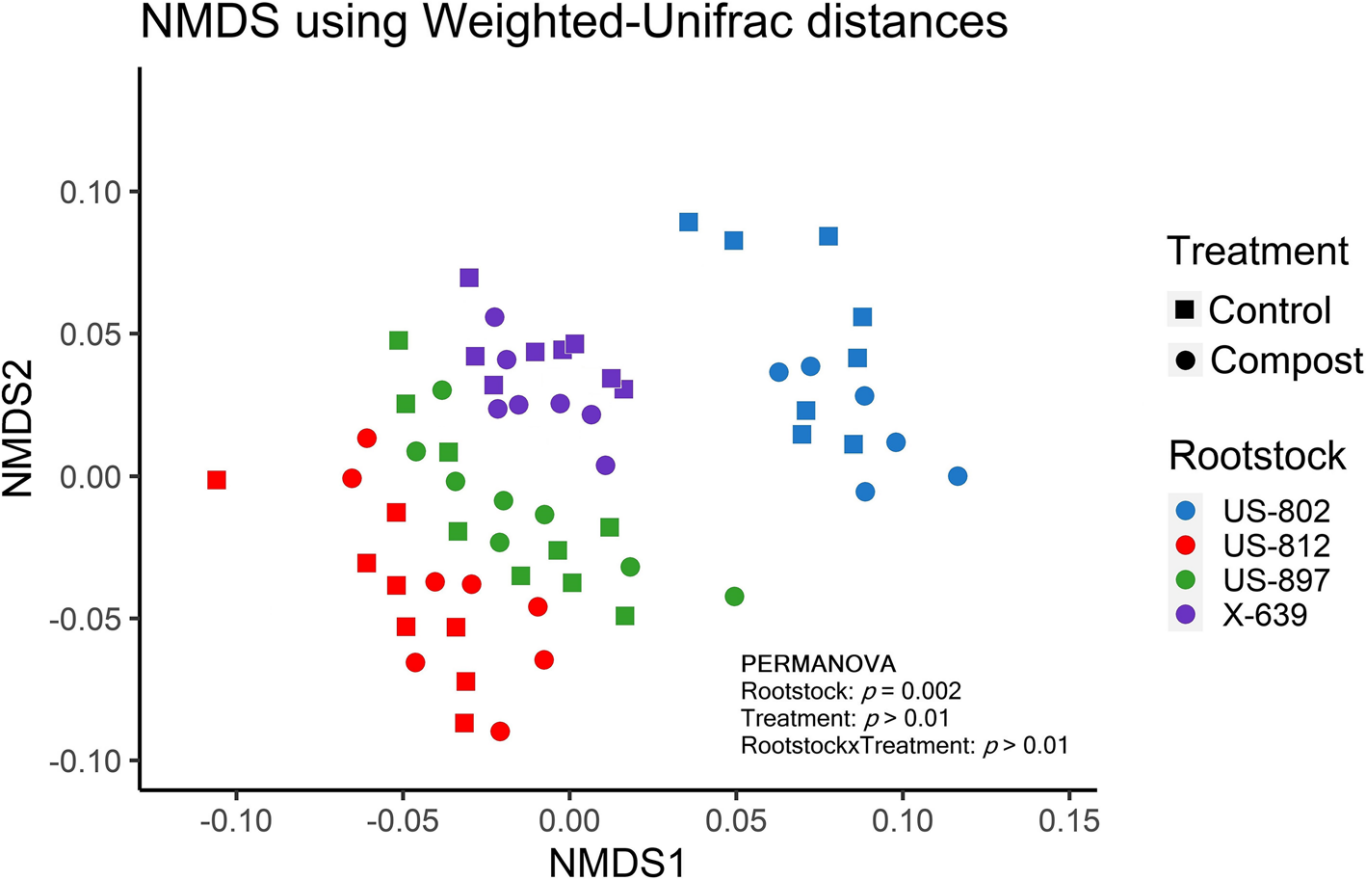
Nonmetric multidimensional scaling (NMDS) plot on Bray-Curtis distance for predicted bacterial functions of active bacterial communities in the rhizosphere of four different rootstocks. Soils were untreated (control) or treated with compost. Differences in community composition between rootstocks, treatments, and their interactions were tested by PERMANOVA. Stress = 0.112

NMDS analysis on Bray-Curtis distance together with a PERMANOVA analysis showed significant differences in the predicted functionality of bacterial communities among rootstocks (*p* = 0.002) and no significant differences between treatments (*p* > 0.01) and rootstock and treatment interaction (*p* > 0.01) (Fig. 5). There were no significant differences in the mean proportion of predicted KEGG categories between rootstocks and treatments, and the categories of energy metabolism and biosynthesis of other secondary metabolites accounted for more than 60% of the predicted functions (Supplementary Fig. S8A). There were only 5 predicted pathways with significant differences between rootstocks and treatments (Supplementary Fig. S8B). Both in the control and treated soils, the pathways of biosynthesis of secondary metabolites and various plant secondary metabolites were significantly more abundant in the rhizobiome of US-802 and X-639 compared to US-812 and US-897. Carbon and nitrogen metabolism pathways were significantly more abundant in the treated soils compared to controls for US-812 and US-897. The pathway involved in tryptophan metabolism had a significantly greater relative abundance in soils treated with compost compared to control for US-802 and X-639 (Supplementary Fig. S8B).

### Relationships between rootstock, compost, MNC, bacterial abundance, alpha- and beta-diversity, and predicted functionality

**Fig. 6:**
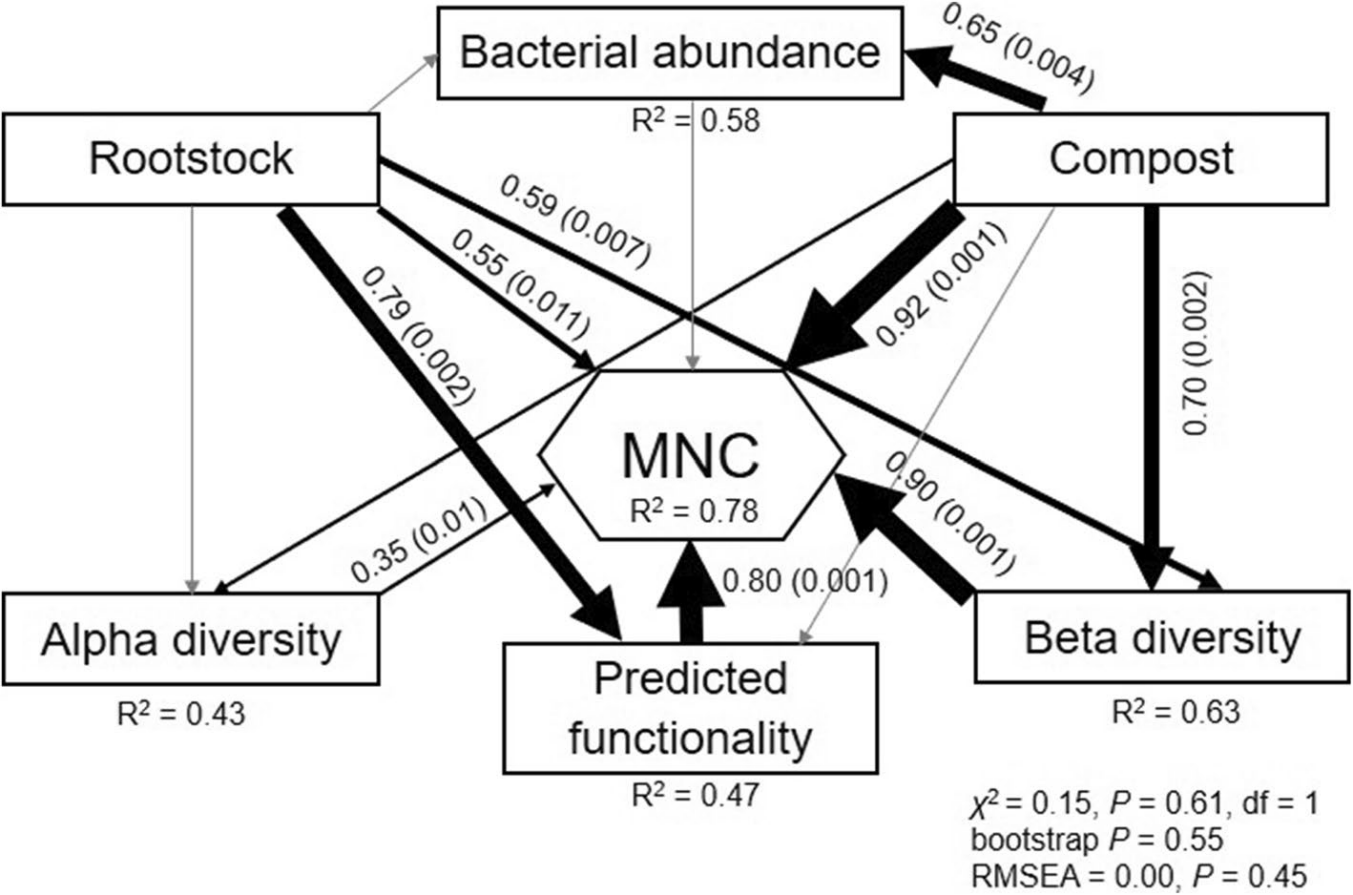
Structural equation model describing the relationships between rootstock, compost, MNC, alpha- and beta-diversity, and predicted functionality. Numbers adjacent to arrows are indicative of the effect size (bootstrap *p*-value) of the relationship. Continuous and dashed arrows indicate positive and negative relationships, respectively. The width of arrows is proportional to the strength of path coefficients. *R*^2^ denotes the proportion of variance explained. Gray lines represent tested, but not significant, paths

Our SEM model explained 78%, 63%, 58%, 47%, and 43% of the variance found in MNC, beta-diversity, bacterial abundance, predicted functionality, and alpha-diversity (Fig. 6). Rootstock and compost had significant positive effects on MNC and beta-diversity, with compost showing stronger impacts (Fig. 6). Rootstock and compost had a significant positive effect on predicted functionality and bacterial abundance, respectively. Compost showed a significant positive effect on bacterial abundance and alpha-diversity (Fig. 6).

### Identification of the active taxonomic and predicted functional core rhizobiome

The taxonomic core rhizobiome was formed by bacterial taxa belonging to the same eleven genera in the control and treated soils and whose relative abundances did not significantly differ between treatments (Supplementary Table S3). The predicted functional core rhizobiome comprised the same thirteen pathways in the control and treated soils (Supplementary Table S4). However, eight of these pathways (tryptophan metabolism, nitrogen metabolism, carbohydrate metabolism, lipid metabolism, metabolism of other amino acids, metabolism of cofactors and vitamins, and xenobiotics and biodegradation metabolism) were significantly more abundant in the treated soils compared to the control (Supplementary Table S4).

## Discussion

We found that the rootstock genotype determined differences in the diversity of active rhizosphere bacterial communities. The rootstock genotype also impacted how compost altered the abundance, diversity, and composition and predicted functions of these active communities. Variations in the active bacterial rhizobiome were strongly linked to root nutrient cycling, and these interactions were root-nutrient- and rootstock-specific. Together, these findings have important agronomic implications as they indicate the potential for agricultural production systems to maximize benefits from rhizobiomes through the choice of selected rootstocks and the application of compost. Direct positive relationships between enriched taxa in treated soils and specific root nutrients were detected which will help identify potentially important taxa for development of agricultural microbiome engineering solutions to improve root nutrient uptake. We also found significant differences in specific predicted functions related to soil nutrient cycling (C, N, and tryptophan metabolisms) in the active bacterial rhizobiome among rootstocks, particularly in soils treated with compost. These results suggest that potential functions of active bacterial rhizobiomes are rootstock-specific rather than redundant among citrus rootstocks.

The rootstock genotype determined differences in bacterial diversity but not community composition of the active bacterial rhizobiome in untreated soils. Previous studies have shown that the root metabolic composition can differ among citrus rootstocks [43–45], which may explain the differences in bacterial diversity among rootstocks in this study. The finding that composition (beta-diversity) remained unchanged among rootstocks in untreated soil suggests no or only a minor influence of rootstocks on the recruitment of bacterial communities in the rhizosphere, which agrees with previously published studies [2, 56].

In addition to influencing the bacterial diversity, the rootstocks used in this study were found to directly influence nutrient cycling through alterations of the active rhizobiome. For example, root N, P, and Ca concentrations were significantly and positively correlated with alpha- and beta-diversity for US-812, but not the other rootstocks. Other root nutrients such as Mg and Cu were positively correlated with alpha- and beta-diversity for all rootstocks, suggesting no rootstock effect but a key role of the active bacterial rhizobiome in driving root nutrient cycling. Magnesium is essential for increasing root system and fruit quality as it promotes the reduction of reactive oxygen species (ROS) [91] and distribution of sugars in the plant [92]. Copper is required for plant growth and development as it is involved in different physiological processes such as photosynthesis, respiration, and ethylene [93].

Regardless of the rootstock genotype, compost application altered the composition of the active bacterial rhizobiome. These compost-driven differences in beta-diversity could be due to the bacterial community shifting from oligotrophic to more copiotrophic bacterial taxa in treated soils, as previously observed in the rhizosphere of apple rootstocks treated with compost [17]. For example, a proliferation of known fast-growing copiotrophic consumers of labile C (e.g., Actinobacteria, *Bacteroidetes*, Chloroflexi, Gemmatimonadetes, and Firmicutes) was observed for all rootstocks in soils treated with compost. These variations in beta-diversity of the active bacterial rhizobiome did not affect core rhizobiome taxa, which suggests that other microbes within the rhizobiome were more responsive to compost application. However, compost application only increased the bacterial abundance and alpha-diversity of the rhizobiome of US-812 and US-897 but not US-802 and X-639. Significant positive correlations between increased bacterial diversity and root multinutrient cycling were detected for US-812 and US-897 rootstocks, suggesting a rootstock-specific impact of compost on the rhizobiome community composition that in turn influences root nutrient cycling. Previous studies have also linked increased soil microbial abundance and diversity to nutrient availability after compost application [51, 52]. Interestingly, US-897 and US-812 are known for their positive influence on fruit quality, whereas US-802 and X-639 are known to produce lower-quality fruit [36, 41]. Whether this effect will be enhanced with compost amendments will need to be investigated as the trees become more mature.

The interaction between citrus rootstocks and compost was a stronger determinant of changes in bacterial abundance, diversity, and community composition of the active bacterial rhizobiome than compost or rootstocks alone. While recent studies have shown that rootstocks [12, 18] and compost [17] can alter microbial diversity and community composition in the rhizobiome of different crops, our results provide strong evidence of compost and rootstock interactions driving changes in the active rhizobiome (alpha- and beta-diversity) with direct impacts on root nutrient availability. Although recent studies have shown that soil microbial diversity promotes multifunctionality in natural ecosystems [74–78], these observations were mainly restricted to nutrient cycling in bulk soils. Here, we expand on those findings by showing that these interactions also occur in the rhizosphere where they can be controlled not only by the rootstock genotype but also the application of compost. For instance, we observed a strong positive correlation between Zn and Mn root concentrations and alpha- and beta-diversity in the rhizobiome of US-812 and US-897 rootstocks. Zn is a micronutrient with a key role in plant defense against pathogens [94], whereas Mn is essential for photosynthesis and a limiting factor for plant growth [95]. Although our results suggest that the rhizobiome composition improves root nutrient cycling, it is uncertain whether this ultimately translates into increased plant growth, crop production, and stress and disease tolerance in the longer term. At the time of the study, no differences in tree growth and health were observed with the compost amendment, but US-897 produced the most fruit in the first year of production, while US-802 was the most vigorous rootstock (data not shown). These results are expected in this early stage of growth, and it may take several years of treatments and until trees reach full maturity before increases in productivity due to any microbe-induced effect may be observed.

Specific active genera in the rhizobiome of composted soils were strongly correlated with root nutrient concentrations. Some of these genera include known plant growth-promoting (PGP) bacteria such as *Bacillus*, *Streptomyces*, *Pseudomonas*, *Mesorhizobium*, *Sphingomonas*, and *Rhizobium* [96, 97] that can solubilize nutrients such as P, S, and Ca and produce diverse phytohormones and siderophores. Although correlation does not imply causation, we found significant associations between several other genera and specific root nutrients in the citrus rhizobiome. For example, members of Acidobacteria were correlated with root Fe, which agrees with several studies reporting that Acidobacteria are avid rhizosphere colonizers and can produce siderophores [98]. We also found strong correlations between members of *Bacteroidetes* and root P concentration which is in line with previous observations of genera assigned to *Bacteroidetes* playing a critical role in solubilization of P in the plant rhizosphere [99]. Cyanobacteria genera such as *Leptolyngbya*, *Nostoc*, *Oscillatoria*, and *Microcoleus* were important for predicting changes in root N, P, and K concentrations which is not surprising as Cyanobacteria are known to improve the availability of N, P, and K through N-fixation and solubilization [100, 101]. We also identified genera assigned to Planctomycetes which were correlated with Fe, which is in accordance with previous studies showing their ability to produce siderophores in soils [102]. Together, this knowledge provides valuable information for selecting candidate taxa for future agricultural microbiome engineering solutions [22, 23]. For example, members of the differentially abundant genera in this study may represent candidate taxa for designing microbial consortia with a potential to serve as biofertilizers [24].

Most predicted functions in the rhizobiome were shared among citrus rootstocks, thus supporting the concept of functional redundancy between plant genotypes of the same crop [103]. However, there were significant differences in functional pathways related to biosynthesis of secondary metabolites and C, N, and tryptophan metabolisms among rootstocks in untreated soils, and compost application increased the abundance of these potential functions; however, the magnitude of the responses was rootstock-specific. Overall, these results are different from those of a recent study examining predicted functions in the rhizobiome of different rootstocks for grapevines [12]. That study found no differences in predicted functions between grapevine rootstocks using the Tax4fun tool to predict functional potential. As recently demonstrated, Tax4fun and PICRUSt2 can lead to differences in predicted bacterial functions which could explain the different results between rhizobiome studies [104]. While Marasco et al. [12] used a DNA-based approach to characterize rhizobiome communities for grapevines, we used RNA-based estimates to predict bacterial functions which can be a more accurate and reliable approach for functional predictions in rhizobiomes [56]. In addition, it cannot be ruled out that genotype-specific root exudates determine rhizobiome functions [2]. Interestingly, we detected eight pathways within the predicted functional core citrus rhizobiome that were more abundant in treated soils compared to the control for all rootstocks. These pathways were related to key functions for plant growth such as N, carbohydrate and lipid metabolisms, and metabolism of cofactors and vitamins. While compost had no impact on the core taxonomic rhizobiome of all rootstocks, it appeared to influence the taxonomic and predicted functional core rhizobiomes. Although PICRUSt2 is frequently used to predict functions of microbial communities and its effectiveness has been established in multiple environmental studies that utilized both amplicon sequencing and metagenome sequencing [71], we acknowledge it has some limitations [105], and other approaches such as shotgun metagenome sequencing can provide more accurate functional profiles of microbiomes. However, our results provide a good starting place for future studies of functional differences between rhizobiomes under the influence of different rootstock genotypes.

## Conclusions

This study showed that the interaction between citrus rootstocks and compost can influence active rhizosphere bacterial communities with impacts on root nutrient concentrations. In particular, the response of the rhizobiome bacterial abundance, diversity, and community composition to compost was rootstock-specific. Specific bacterial taxa therefore appear to be driving changes in root nutrient concentrations in the active rhizobiome of different citrus rootstocks. Whether rootstock genotype-specific impacts on rhizosphere microbes also determine variations in nutrient concentration in rhizosphere soil and other parts of the tree (e.g., leaves and trunk) should be explored in future studies. In addition, several potential functions of active bacterial rhizobiomes recruited by different citrus rootstocks did not appear to be redundant but rather rootstock-specific. Longer-term studies will determine to what extent rhizobiome alterations impact aboveground traits, especially tree growth and productivity but also resilience to HLB. The study of root exudate composition could also help identify associations of individual taxa with specific root exudate compounds and provide an understanding of how rootstocks and compost control these relationships.

## Supplementary Information

**Additional file 1:** **Fig. S1.** Schematic diagram of the field study illustrating the experimental design (A); an untreated control plot (left) and a compost-treated plot (right) – trees are arranged in two rows on raised beds separated by furrows for drainage (B); a grafted citrus tree composed of scion and rootstock that are united at the graft union (C). **Fig. S2.** A priori generic structural equation model (SEM) used in this study. The numbers in the arrows denote example references used to support our predictions (see References section). **Fig. S3.** Root nutrient content of citrus trees on four different rootstocks. Soils were untreated (control) or treated with compost. Different letters above the bars indicate significant differences between rootstocks and treatments (linear mixed-effect model and Tukey's HSD; *n* = 8; *, *p* ≤ 0.05; **, *p* ≤ 0.01; ***, *p* ≤ 0.001). Values are expressed as mean with standard error. **Fig. S4.** Total abundance of active bacterial communities in the rhizosphere of citrus trees on four different rootstocks. Soils were untreated (control) or treated with compost. Different letters above the bars indicate significant differences between rootstocks and treatments (linear mixed-effect model and Tukey's HSD, *n* = 8; *, *p* ≤ 0.05; **, *p* ≤ 0.01; ***, *p* ≤ 0.001). Values are expressed as mean with standard error. **Fig. S5.** Relative abundance of bacterial ASVs at the phylum taxonomic level in the rhizosphere of four different rootstocks. Soils were untreated (control) or treated with compost. **Fig. S6.** Differentially abundant ASVs at the genus taxonomic level between compost and control treatments for each rootstock. The fold change is shown on the X axis and genera are listed on the Y axis. Each colored dot represents an ASV that was identified by DESeq2 analysis as significantly differentially abundant (*p* ≤ 0.05). **Fig. S7.** Heatmaps of Spearman correlation coefficients between bacterial alpha (A) and beta (B) diversity and root nutrients for each rootstock. The shading from blue to red represents low-to-high positive correlation. *, *p* ≤ 0.05; **, *p* ≤ 0.01; ***, *p* ≤ 0.001. **Fig. S8.** Mean proportion of predicted KEGG categories (A) and pathways (B) in the rhizosphere of citrus trees on four different rootstocks. Soils were untreated (control) or treated with compost. For each row, different letters indicate significant differences between treatments and rootstocks (Tukey's HSD, *p* < 0.05; *n* = 8). **Table S1.** Rootstocks used in this study and their parentage. **Table S2.** Significance and similarity using the non-parametric multivariate ANOSIM statistical method. Numbers in bold indicate significant effect at *p* < 0.05. R values close to 1 indicate dissimilarity between treatments. **Table S3.** ASVs (at the genus level) present in at least 75% of the samples in the control and treated soils identified as the active taxonomic core rhizobiome and their relative abundances. For each row, different letters between treatments indicate significant according to the Welch’s t-test and Benjamini–Hochberg FDR multiple test correction (*p* < 0.05). Table S4. KEGG pathways present in at least 75% of the samples in the control and treated soils identified as the active functional core rhizobiome and their relative abundances. For each row, different letters between treatments indicate significant according to the Welch’s t-test and Benjamini–Hochberg FDR multiple test correction (*p* < 0.05)

## Data Availability

Raw sequencing reads are available in in NCBI’s Sequence Read Archive under BioProject PRJNA837574.

## References

[1] Bais HP, Weir TL, Perry LG, Gilroy S, Vivanco JM (2006). The role of root exudates in rhizosphere interactions with plants and other organisms. Annual Reviews..

[2] Bulgarelli D, Schlaeppi K, Spaepen S, Van Themaat EVL, Schulze-Lefert P (2013). Structure and functions of the bacterial microbiota of plants. Annual Reviews..

[3] Edwards J, Johnson C, Santos-Medellín C, Lurie E, Podishetty NK, Bhatnagar S (2015). Structure, variation, and assembly of the root-associated microbiomes of rice. Proc Natl Acad Sci U S A..

[4] Bulgarelli D, Garrido-Oter R, Münch PC, Weiman A, Dröge J, Pan Y (2015). Structure and function of the bacterial root microbiota in wild and domesticated barley. Cell Host Microbe. Cell Press..

[5] Lareen A, Burton F, Schäfer P (2016). Plant root-microbe communication in shaping root microbiomes. Plant Mol Biol..

[6] Hamonts K, Trivedi P, Garg A, Janitz C, Grinyer J, Holford P (2018). Field study reveals core plant microbiota and relative importance of their drivers. Environ Microbiol..

[7] Goldschmidt EE (2014). Plant grafting: new mechanisms, evolutionary implications. Front Plant Sci..

[8] Albrecht U, Zekri M, Williamson J. Citrus propagation. https://edis.ifas.ufl.edu/publication/HS1309 (2021). Accessed 31 May 2022.

[9] Bowman KD, McCollum G, Albrecht U (2021). SuperSour: a new strategy for breeding superior citrus rootstocks. Front Plant Sci..

[10] Mudge K, Janick J, Scofield S, Goldschmidt EE (2009). A history of grafting. Hortic Rev..

[11] D´Amico F, Candela M, Turroni S, Biagi E, Brigidi P, Bega A (2018). The rootstock regulates microbiome diversity in root and rhizosphere compartments *Vitis vinifera* cultivar lambrusco. Front Microbiol.

[12] Marasco R, Rolli E, Fusi M, Michoud G, Daffonchio D (2018). Grapevine rootstocks shape underground bacterial microbiome and networking but not potential functionality. Microbiome..

[13] Dries L, Bussotti S, Pozzi C, Kunz R, Schnell S, Löhnertz O (2021). Rootstocks shape their microbiome—bacterial communities in the rhizosphere of different grapevine rootstocks. Microorganisms..

[14] Vink SN, Dini-Andreote F, Höfle R, Kicherer A, Salles JF (2021). Interactive effects of scion and rootstock genotypes on the root microbiome of grapevines  (*Vitis* spp. L.). Appl Sci.

[15] Liu J, Abdelfattah A, Norelli J, Burchard E, Schena L, Droby S (2018). Apple endophytic microbiota of different rootstock/scion combinations suggests a genotype-specific influence. Microbiome..

[16] van Horn C, Somera TS, Mazzola M (2021). Comparative analysis of the rhizosphere and endophytic microbiomes across apple rootstock genotypes in replant orchard soils. Phytobiomes J..

[17] Sharaf H, Thompson AA, Williams MA, Peck GM (2021). Compost applications increase bacterial community diversity in the apple rhizosphere. Soil Sci Soc Am J..

[18] Poudel R, Jumpponen A, Kennelly MM, Rivard CL, Gomez-Montano L, Garrett KA (2019). Rootstocks shape the rhizobiome: rhizosphere and endosphere bacterial communities in the grafted tomato system. Appl Environ Microbiol..

[19] Bonito G, Reynolds H, Robeson MS, Nelson J, Hodkinson BP, Tuskan G (2014). Plant host and soil origin influence fungal and bacterial assemblages in the roots of woody plants. Mol Ecol..

[20] Veach AM, Morris R, Yip DZ, Yang ZK, Engle NL, Cregger MA (2019). Rhizosphere microbiomes diverge among *Populus trichocarpa* plant-host genotypes and chemotypes, but it depends on soil origin. Microbiome.

[21] Liu S, He F, Kuzyakov Y, Xiao H, Hoang DTT, Pu S (2022). Nutrients in the rhizosphere: a meta-analysis of content, availability, and influencing factors. Sci Total Environ..

[22] Mueller UG, Sachs JL (2015). Engineering microbiomes to improve plant and animal health. Trends Microbiol..

[23] Panke-Buisse K, Poole AC, Goodrich JK, Ley RE, Kao-Kniffin J (2015). Selection on soil microbiomes reveals reproducible impacts on plant function. ISME J..

[24] Poudel R, Jumpponen A, Schlatter DC, Paulitz TC, McSpadden-Gardener BB, Kinkel LL (2016). Microbiome networks: a systems framework for identifying candidate microbial assemblages for disease management. Phytopathology.

[25] Mahmud K, Missaoui A, Lee K, Ghimire B, Presley HW, Makaju S (2021). Rhizosphere microbiome manipulation for sustainable crop production. Curr Plant Biol..

[26] Gottwald TR, Graça JV da, Bassanezi RB. Citrus huanglongbing: the pathogen and its impact. Plant Health Prog. 2007;8:1–31.

[27] Wang N, Stelinski LL, Pelz-Stelinski KS, Graham JH, Zhang Y (2017). Tale of the huanglongbing disease pyramid in the context of the citrus microbiome. Phytopathology..

[28] Graham J, Gottwald T, Setamou M (2020). Status of huanglongbing (HLB) outbreaks in Florida. California and Texas. Trop Plant Pathol..

[29] Bowman KD, Joubert J, Talon M, Caruso M, Gmitter FG (2020). Citrus rootstocks. The genus Citrus.

[30] Zambon FT, Kadyampakeni DM, Grosser JW (2019). Ground application of overdoses of manganese have a therapeutic effect on sweet orange trees infected with *Candidatus* liberibacter asiaticus. HortScience..

[31] Abobatta WF, El-Azazy AM (2020). Role of organic and biofertilizers in citrus orchards. Aswan Univ J Environ Stud..

[32] Castellano-Hinojosa A, Meyering B, Nuzzo A, Strauss SL, Albrecht U (2021). Effect of plant biostimulants on root and plant health and the rhizosphere microbiome of citrus trees in huanglongbing-endemic conditions. Trees - Struct Funct..

[33] Wang N, Pierson EA, Setubal JC, Xu J, Levy JG, Zhang Y (2017). The *Candidatus* Liberibacter–host interface: insights into pathogenesis mechanisms and disease control. Annual Reviews..

[34] Song C, Zhu F, Carrión VJ, Cordovez V (2020). Beyond plant microbiome composition: exploiting microbial functions and plant traits via integrated approaches. Front Bioeng Biotechnol..

[35] Zhang Y, Trivedi P, Xu J, Roper C, Wang N (2021). The citrus microbiome: from structure and function to microbiome engineering and beyond. Phytobiomes.

[36] Castle WS (2010). A career perspective on citrus rootstocks, their development, and commercialization. HortScience..

[37] Wutscher HK. Citrus Rootstocks. Hortic Rev. 1979;1:237–69.

[38] Bowman KD, McCollum G, Albrecht U (2016). Performance of ‘Valencia’ orange (Citrus sinensis [L.] Osbeck) on 17 rootstocks in a trial severely affected by huanglongbing. Sci Hortic.

[39] Dubey AK, Sharma RM (2016). Effect of rootstocks on tree growth, yield, quality and leaf mineral composition of lemon  (*Citrus limon* (L.) Burm.). Sci Hortic.

[40] Albrecht U, Fiehn O, Bowman KD (2016). Metabolic variations in different citrus rootstock cultivars associated with different responses to huanglongbing. Plant Physiol Biochem..

[41] Kunwar S, Grosser J, Gmitter FG, Castle WS, Albrecht U (2021). Field performance of ‘hamlin’ orange trees grown on various rootstocks in huanglongbing-endemic conditions. HortScience..

[42] Kunwar S, Meyering B, Grosser J, Gmitter FG, Castle WS, Albrecht U (2023). Field performance of ‘Valencia’ orange trees on diploid and tetraploid rootstocks in different huanglongbing-endemic growing environments. Sci Hortic..

[43] Dong X, Liu G, Wu X, Lu X, Yan L, Muhammad R (2016). Different metabolite profile and metabolic pathway with leaves and roots in response to boron deficiency at the initial stage of citrus rootstock growth. Plant Physiol Biochem..

[44] Albrecht U, Tripathi I, Kim H, Bowman KD (2019). Rootstock effects on metabolite composition in leaves and roots of young navel orange  (*Citrus sinensis* L. Osbeck) and pummelo (C. grandis L. Osbeck) trees. Trees Struct Funct.

[45] Albrecht U, Tripathi I, Bowman KD (2020). Rootstock influences the metabolic response to Candidatus Liberibacter asiaticus in grafted sweet orange trees. Trees - Struct Funct..

[46] United States Department of Agriculture (USDA) Economic research service https://www.ers.usda.gov/topics/crops/fruit-tree-nuts/ (2022). Accessed 31 May 2022.

[47] Obreza TA, Collins ME. Common soils used for citrus production in Florida. 2008. https://ufdcimages.uflib.ufl.edu/IR/00/00/31/34/00001/SS40300.pdf. Accessed 31 May 2022.

[48] Johnson EG, Wu J, Bright DB, Graham JH (2014). Association of ‘Candidatus Liberibacter asiaticus’ root infection, but not phloem plugging with root loss on huanglongbing-affected trees prior to appearance of foliar symptoms. Plant Pathol..

[49] Ozores-Hampton M, Stansly PA, McSorley R, Obreza TA (2005). Effects of long-term organic amendments and soil solarization on pepper and watermelon growth, yield, and soil fertility. HortScience..

[50] Lehmann J, Rillig MC, Thies J, Masiello CA, Hockaday WC, Crowley D (2011). Biochar effects on soil biota – a review. Soil Biol. Biochem..

[51] Strauss SL, Stover JK, Kluepfel DA (2015). Impact of biological amendments on Agrobacterium tumefaciens survival in soil. Appl Soil Ecol. Elsevier.

[52] Thompson AA, Williams MA, Peck GM (2019). Compost and Geneva® series rootstocks increase young ‘Gala’ apple tree growth and change root-zone microbial communities. Sci Hortic..

[53] Pérez-Piqueres A, Edel-Hermann V, Alabouvette C, Steinberg C (2006). Response of soil microbial communities to compost amendments. Soil Biol Biochem..

[54] Van Elsas JD, Chiurazzi M, Mallon CA, Elhottova D, Krištůfek V, Salles JF (2012). Microbial diversity determines the invasion of soil by a bacterial pathogen. Proc Natl Acad Sci U S A..

[55] Jousset A, Bienhold C, Chatzinotas A, Gallien L, Gobet A, Kurm V (2017). Where less may be more: how the rare biosphere pulls ecosystems strings. ISME J..

[56] Vieira S, Sikorski J, Dietz S, Herz K, Schrumpf M, Bruelheide H (2019). Drivers of the composition of active rhizosphere bacterial communities in temperate grasslands. ISME J.

[57] Carini P, Marsden PJ, Leff JW, Morgan EE, Strickland MS, Fierer N (2016). Relic DNA is abundant in soil and obscures estimates of soil microbial diversity. Nat Microbiol..

[58] Gkarmiri K, Mahmood S, Ekblad A, Alström S, Högberg N, Finlay R (2017). Identifying the active microbiome associated with roots and rhizosphere soil of oilseed rape. Appl Environ Microbiol.

[59] United States Department of Agriculture (USDA), Soil survey staff illustrated guide to soil taxonomy. https://www.nrcs.usda.gov/wps/portal/nrcs/detail/soils/survey/class/taxonomy/?cid=nrcs142p2_053580 (2015). Accessed 31 May 2022.

[60] Mylavarapu R, Harris W, Hochmuth G. Agricultural soils of Florida. https://edis.ifas.ufl.edu/ss655 (2016). Accessed 31 May 2022.

[61] Donohue SJ, Aho DW, Plank CO (1992). Determination of P, K, Ca, Mg, Mn, Fe, Al, B, Cu, and Zn in plant tissue by inductively coupled plasma (ICP) emission spectroscopy. Plant analysis reference procedures for the Southern Region of the United States, Southern Cooperative Series Bulletin 368.

[62] Castellano-Hinojosa A, Martens-Habbena W, Strauss SL (2022). Cover crop composition drives changes in the abundance and diversity of nitrifiers and denitrifiers in citrus orchards with critical effects on N_2_O emissions. Geoderma..

[63] Apprill A, McNally S, Parsons R, Weber L (2015). Minor revision to V4 region SSU rRNA 806R gene primer greatly increases detection of SAR11 bacterioplankton. Aquat. Microb. Ecol..

[64] Castellano-Hinojosa A, Strauss SL (2021). Insights into the taxonomic and functional characterization of agricultural crop core rhizobiomes and their potential microbial drivers. Sci Reports.

[65] Callahan BJ, McMurdie PJ, Rosen MJ, Han AW, Johnson AJA, Holmes SP (2016). DADA2: high-resolution sample inference from Illumina amplicon data. Nat Methods..

[66] Quast C, Pruesse E, Yilmaz P, Gerken J, Schweer T, Yarza P (2013). The SILVA ribosomal RNA gene database project: improved data processing and web-based tools. Nucleic Acids Res..

[67] Bokulich NA, Kaehler BD, Rideout JR, Dillon M, Bolyen E, Knight R, et al. Optimizing taxonomic classification of marker-gene amplicon sequences with QIIME 2’s q2-feature-classifier plugin. Microbiome. 2018;6:1–17. 10.1186/s40168-018-0470-z PMC5956843 29773078

[68] McMurdie PJ, Holmes S (2013). phyloseq: an R package for reproducible interactive analysis and graphics of microbiome census data. PLoS One..

[69] McMurdie PJ, Holmes S (2014). Waste not, want not: why rarefying microbiome data is inadmissible. PLOS Comput Biol..

[70] Love MI, Huber W, Anders S (2014). Moderated estimation of fold change and dispersion for RNA-seq data with DESeq2. Genome Biol..

[71] Douglas GM, Maffei VJ, Zaneveld JR, Yurgel SN, Brown JR, Taylor CM (2020). PICRUSt2 for prediction of metagenome functions. Nat Biotechnol..

[72] Benjamini Y, Hochberg Y (1995). Controlling the false discovery rate: a practical and powerful approach to multiple testing. J. R. Stat. Soc. B..

[73] Bardgett R, van der Putten W (2014). Belowground biodiversity and ecosystem functioning. Nature..

[74] Delgado-Baquerizo M, Maestre FT, Reich PB, Jeffries TC, Gaitan JJ, Encinar D (2015). Microbial diversity drives multifunctionality in terrestrial ecosystems. Nat Commun..

[75] Jing X, Sanders NJ, Shi Y, Chu H, Classen AT, Zhao K (2015). The links between ecosystem multifunctionality and above- and belowground biodiversity are mediated by climate. Nat Commun..

[76] Jiao S, Chen W, Wang J, Du N, Li Q, Wei G (2018). Soil microbiomes with distinct assemblies through vertical soil profiles drive the cycling of multiple nutrients in reforested ecosystems. Microbiome..

[77] Jiao S, Xu Y, Zhang J, Hao X, Lu Y (2019). Core microbiota in agricultural soils and their potential associations with nutrient cycling. mSystems.

[78] Jiao S, Peng Z, Qi J, Gao J, Wei G (2021). Linking bacterial-fungal relationships to microbial diversity and soil nutrient cycling. mSystems.

[79] Mattos DJ, Kadyampakeni DM, Oliver AQ, Boaretto RM, Morgan KT, Talon M, Caruso M, Gmitter FF (2020). Quaggio JA (2020) Soil and nutrition interactions. The genus citrus.

[80] Walker J (1991). Biogeochemical cycles. Science..

[81] White PJ, Broadley MR (2003). Calcium in plants. Ann Bot..

[82] Narayan OP, Kumar P, Yadav B, Dua M, Johri AK. Sulfur nutrition and its role in plant growth and development. Plant Signal Behav. 2022;2030082. 10.1080/15592324.2022.2030082 PMC10730164 35129079

[83] Maestre FT, Quero JL, Gotelli NJ, Escudero A, Ochoa V, Delgado-Baquerizo M (2012). Plant species richness and ecosystem multifunctionality in global drylands..

[84] Kanehisa M, Goto S, Sato Y, Furumichi M, Tanabe M (2012). KEGG for integration and interpretation of large-scale molecular data sets. Nucleic Acids Res..

[85] Xu J, Zhang Y, Zhang P, Trivedi P, Riera N, Wang Y (2018). The structure and function of the global citrus rhizosphere microbiome. Nat Commun..

[86] Groemping U, Matthias L (2013). Relaimpo: Relative importance for linear regression in R: the package relaimpo. J. Stat. Softw..

[87] Grace JB (2006). Structural equation modeling and natural systems.

[88] Whitford WG (2002). Ecology of desert systems London.

[89] Schermelleh-Engel K, Moosbrugger H, Müller H (2003). Evaluating the fit of structural equation models: tests of significance and descriptive goodness-of-fit measures. Methods Psychol. Res..

[90] Parks DH, Tyson GW, Hugenholtz P, Beiko RG (2014). STAMP: statistical analysis of taxonomic and functional profiles. Bioinformatics..

[91] Sewelam N, Kazan K, Schenk PM (2016). Global plant stress signaling: reactive oxygen species at the cross-road. Front Plant Sci.

[92] Morton AR, Trolove SN, Kerckhoffs LHJ (2008). Magnesium deficiency in citrus grown in the Gisborne District of New Zealand. N. Z. J. Crop Hortic. Sci..

[93] Yuan HM, Xu HH, Liu WC, Lu YT (2013). Copper regulates primary root elongation through PIN1-mediated auxin redistribution. Plant Cell Physiol..

[94] Cabot C, Martos S, Llugany M, Gallego B, Tolrà R, Poschenrieder C (2019). A role for zinc in plant defense against pathogens and herbivores. Front Plant Sci..

[95] Alejandro S, Höller S, Meier B, Peiter E (2020). Manganese in plants: from acquisition to subcellular allocation. Front Plant Sci..

[96] Glick BR (2012). Plant growth-promoting bacteria: mechanisms and applications. Scientifica.

[97] Chandran H, Meena M, Swapnil P (2021). Plant growth-promoting rhizobacteria as a green alternative for sustainable agriculture. Sustainability.

[98] Kalam S, Basu A, Ahmad I, Sayyed RZ, El-Enshasy HA, Dailin DJ (2020). Recent understanding of soil Acidobacteria and their ecological significance: a critical review. Front Microbiol..

[99] Lidbury IDEA, Borsetto C, Murphy ARJ, Bottrill A, Jones AME, Bending GD (2020). Niche-adaptation in plant-associated Bacteroidetes favours specialisation in organic phosphorus mineralisation. ISME J..

[100] Jan Z, Ali S, Sultan T, Wasiullah W, Ahmad W (2017). The role of cyanobacteria in availability of major plant nutrients and soil organic matter to rice crop under saline soil condition. Sarhad J Agric..

[101] Santini G, Biondi N, Rodolfi L, Tredici MR (2021). Plant biostimulants from cyanobacteria: an emerging strategy to improve yields and sustainability in agriculture. Plants.

[102] Kaboré OD, Godreuil S, Drancourt M (2020). Planctomycetes as host-associated bacteria: a perspective that holds promise for their future isolations, by mimicking their native environmental niches in clinical microbiology laboratories. Front Cell Infect Microbiol..

[103] Mendes LW, Kuramae EE, Navarrete AA, Van Veen JA, Tsai SM (2014). Taxonomical and functional microbial community selection in soybean rhizosphere. ISME J..

[104] Toole DR, Zhao J, Martens-Habbena W, Strauss SL (2021). Bacterial functional prediction tools detect but underestimate metabolic diversity compared to shotgun metagenomics in southwest Florida soils. Appl Soil Ecol..

[105] Sun S, Jones RB, Fodor AA (2020). Inference-based accuracy of metagenome prediction tools varies across sample types and functional categories. Microbiome..

