# Supplementary material for: Interactions between rootstocks and compost influence the active rhizosphere bacterial communities in citrus
Source: Microbiome. 2023 Apr 20;11:79. doi: 10.1186/s40168-023-01524-y (PMC10116748; doi:10.1186/s40168-023-01524-y)
Supplement: Supplementary file 2 — Additional file 1: Fig. S1. Schematic diagram of the field study illustrating the experimental design (A); an untreated control plot (left) and a compost-treated plot (right) – trees are arranged in two rows on raised beds separated by furrows for drainage (B); a grafted citrus tree composed of scion and rootstock that are united at the graft union (C). Fig. S2. A priori generic structural equation model (SEM) used in this study. The numbers in the arrows denote example references used to support our predictions (see References section). Fig. S3. Root nutrient content of citrus trees on four different rootstocks. Soils were untreated (control) or treated with compost. Different letters above the bars indicate significant differences between rootstocks and treatments (linear mixed-effect model and Tukey's HSD; n = 8; *, p ≤ 0.05; **, p ≤ 0.01; ***, p ≤ 0.001). Values are expressed as mean with standard error. Fig. S4. Total abundance of active bacterial communities in the rhizosphere of citrus trees on four different rootstocks. Soils were untreated (control) or treated with compost. Different letters above the bars indicate significant differences between rootstocks and treatments (linear mixed-effect model and Tukey's HSD, n = 8; *, p ≤ 0.05; **, p ≤ 0.01; ***, p ≤ 0.001). Values are expressed as mean with standard error. Fig. S5. Relative abundance of bacterial ASVs at the phylum taxonomic level in the rhizosphere of four different rootstocks. Soils were untreated (control) or treated with compost. Fig. S6. Differentially abundant ASVs at the genus taxonomic level between compost and control treatments for each rootstock. The fold change is shown on the X axis and genera are listed on the Y axis. Each colored dot represents an ASV that was identified by DESeq2 analysis as significantly differentially abundant (p ≤ 0.05). Fig. S7. Heatmaps of Spearman correlation coefficients between bacterial alpha (A) and beta (B) diversity and root nutrients for each rootstock. [file 40168_2023_1524_MOESM1_ESM.docx]

## **Supplementary Information**

Title: Interactions between rootstocks and compost influence the active rhizosphere bacterial communities in citrus

Authors: Antonio Castellano-Hinojosa^1*^, Ute Albrecht^2^, Sarah L. Strauss^1*^

Affiliations:

^1^Southwest Florida Research and Education Center, Department of Soil, Water, and Ecosystem Sciences, Institute of Food and Agricultural Sciences, University of Florida, 2685 State Rd 29N, Immokalee, FL, 34142, USA

^2^ Southwest Florida Research and Education Center, Department of Horticultural Sciences, Institute of Food and Agricultural Sciences, University of Florida, 2685 State Rd 29N, Immokalee, FL, 34142, USA

*Correspondence:

Antonio Castellano-Hinojosa and Sarah L. Strauss

^1^Southwest Florida Research and Education Center, Department of Soil, Water, and Ecosystem Sciences, Institute of Food and Agricultural Sciences, University of Florida, 2685 State Rd 29N, Immokalee, FL, 34142, USA

Email: antonio.castella@ufl.edu; strauss@ufl.edu

**Table S1.** Rootstocks used in this study and their parentage

| **Rootstock** | **Parentage** |
| --- | --- |
| US-802 | *C. maxima* ‘Siamese’ x *Poncirus trifoliata* ‘Gotha Road’ |
| US-812 | *C. reticulata* ‘Sunki’ x *P. trifoliata* ‘Benecke’ |
| US-897 | *C. reticulata* ‘Cleopatra’ x *P. trifoliata* ‘Flying Dragon’ |
| X-639 | *C. reticulata* ‘Cleopatra’ x *P. trifoliata* ‘Rubidoux’ |

**Table S2.** Significance and similarity using the non-parametric multivariate ANOSIM statistical method. Numbers in bold indicate significant effect at *p* < 0.05. R values close to 1 indicate dissimilarity between treatments.

| Comparison (rootstocks) | ANOSIM | |  |
| --- | --- | --- | --- |
|  | *R* | *p* |  |
| Control | | | |
| US-802 vs. US-812 | 0.206 | 0.259 |  |
| US-802 vs. US-897 | 0.294 | 0.151 |  |
| US-802 vs. X-639 | 0.322 | 0.155 |  |
| US-812 vs. US-897 | 0.346 | 0.195 |  |
| US-812 vs. X-639 | 0.281 | 0.264 |  |
| US-897 vs. X-639 | 0.210 | 0.173 |  |
| Compost | | | |
| US-802 vs. US-812 | **0.916** | **0.001** |  |
| US-802 vs. US-897 | **0.914** | **0.003** |  |
| US-802 vs. X-639 | **0.882** | **0.001** |  |
| US-812 vs. US-897 | **0.886** | **0.005** |  |
| US-812 vs. X-639 | 0.784 | 0.066 |  |
| US-897 vs. X-639 | 0.730 | 0.054 |  |

**Table S3.** ASVs (at the genus level) present in at least 75% of the samples in the control and treated soils identified as the active taxonomic core rhizobiome and their relative abundances. For each row, different letters between treatments indicate significant according to the Welch’s t-test and Benjamini–Hochberg FDR multiple test correction (p < 0.05)

| Active core taxonomic rhizobiome | | | |
| --- | --- | --- | --- |
| Control | Relative abundance (%) | Compost | Relative abundance (%) |
| *Agrobacterium* | 0.7 ± 0.1a | *Agrobacterium* | 0.8 ± 0.2a |
| *Bradyrhizobium* | 4.1 ± 0.3a | *Bradyrhizobium* | 4.6 ± 0.3a |
| *Burkholderia* | 2.4 ± 0.3a | *Burkholderia* | 2.2 ± 0.2a |
| *Cupriavidus* | 3.1 ± 0.4a | *Cupriavidus* | 3.5 ± 0.6a |
| *Mesorhizobium* | 0.9 ± 0.2a | *Mesorhizobium* | 1.2 ± 0.4a |
| *Micromonospora* | 0.5 ± 0.2a | *Micromonospora* | 0.7 ± 0.2a |
| *Pseudomonas* | 0.6 ± 0.2a | *Pseudomonas* | 0.8 ± 0.3a |
| *Phyllobacterium* | 0.6 ± 0.2a | *Phyllobacterium* | 0.7 ± 0.2a |
| *Rhizobium* | 1.9 ± 0.3a | *Rhizobium* | 1.5 ± 0.3a |
| *Serratia* | 0.8 ± 0.2a | *Serratia* | 0.9 ± 0.2a |
| *Sphingomonas* | 0.9 ± 0.3a | *Sphingomonas* | 1.2 ± 0.3a |

**Table S4.** KEGG pathways present in at least 75% of the samples in the control and treated soils identified as the active functional core rhizobiome and their relative abundances. For each row, different letters between treatments indicate significant according to the Welch’s t-test and Benjamini–Hochberg FDR multiple test correction (p < 0.05)

| Active core functional rhizobiome | | | |
| --- | --- | --- | --- |
| Control | Relative abundance (%) | Compost | Relative abundance (%) |
| Biofilm formation | 1.6 ± 0.3a | Biofilm formation | 1.4 ± 0.2a |
| Bacterial chemotaxis | 1.9 ± 0.4a | Bacterial chemotaxis | 1.5 ± 0.3a |
| ABC transporters | 1.9 ± 0.3a | ABC transporters | 1.9 ± 0.2a |
| Bacterial secretion system | 1.9 ± 0.4a | Bacterial secretion system | 1.7 ± 0.3a |
| Two-component system | 1.7 ± 0.3a | Two-component system | 1.9 ± 0.4a |
| Quorum sensing | 1.5 ± 0.3a | Quorum sensing | 1.4 ± 0.3a |
| Biosynthesis of plant hormones | 2.0 ± 0.4a | Biosynthesis of plant hormones | 1.9 ± 0.2a |
| Biosynthesis of plant secondary metabolites | 1.9 ± 0.3a | Biosynthesis of plant secondary metabolites | 1.6 ± 0.3a |
| Diterpenoid biosynthesis | 0.6 ± 0.2a | Diterpenoid biosynthesis | 0.5 ± 0.2a |
| Tryptophan metabolism | 0.6 ± 0.1b | Tryptophan metabolism | 1.8 ± 0.3a |
| Nitrogen metabolism | 3.5 ± 0.5b | Nitrogen metabolism | 4.9 ± 0.4a |
| Carbohydrate metabolism | 7.1 ± 0.5b | Carbohydrate metabolism | 8.5 ± 0.7a |
| Lipid metabolism | 1.7 ± 0.3b | Lipid metabolism | 3.5 ± 0.3a |
| Cell motility | 1.5 ± 0.4a | Cell motility | 1.7 ± 0.2a |
| Metabolism of other amino acids | 1.6 ± 0.3b | Metabolism of other amino acids | 2.9 ± 0.5a |
| Membrane transport | 2.5 ± 0.6a | Membrane transport | 2.6 ± 0.4a |
| Metabolism of cofactors and vitamins | 0.9 ± 0.1b | Metabolism of cofactors and vitamins | 1.5 ± 0.3a |
| Transcription | 0.7 ± 0.2a | Transcription | 0.5 ± 0.2a |
| Xenobiotics and biodegradation metabolism | 0.5 ± 0.2b | Xenobiotics and biodegradation metabolism | 1.6 ± 0.3a |

A.

B.

**
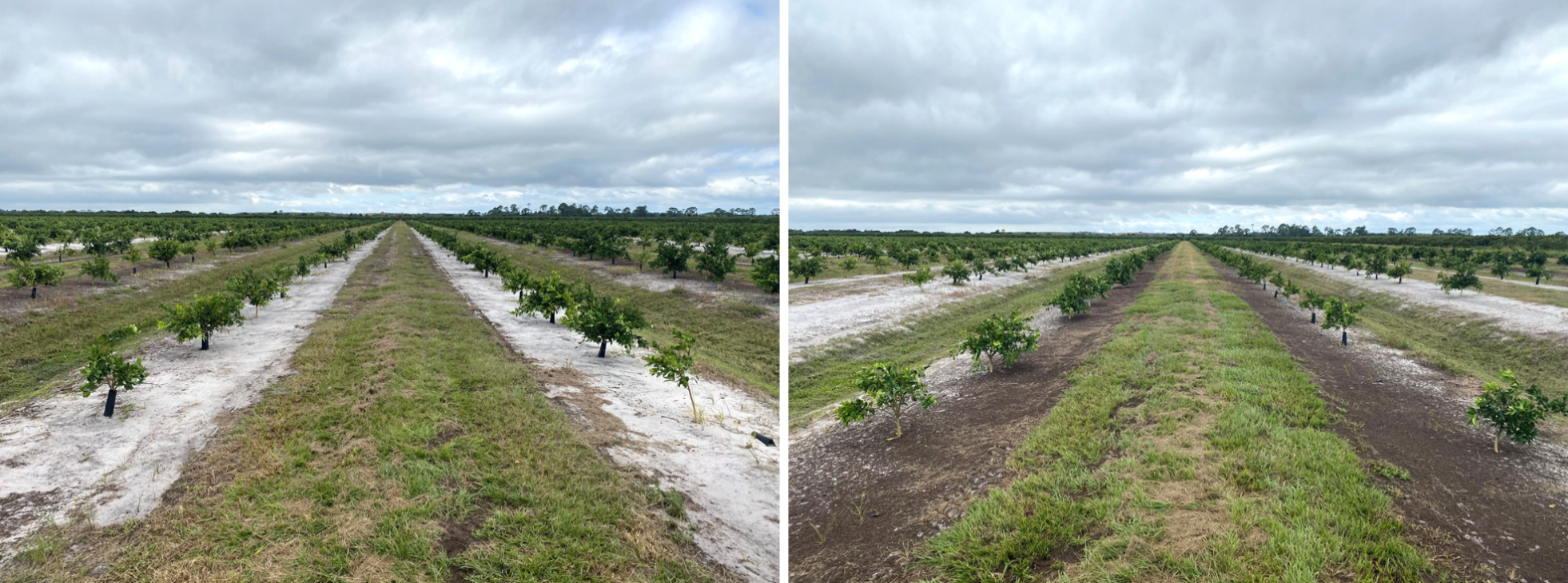
**

C.


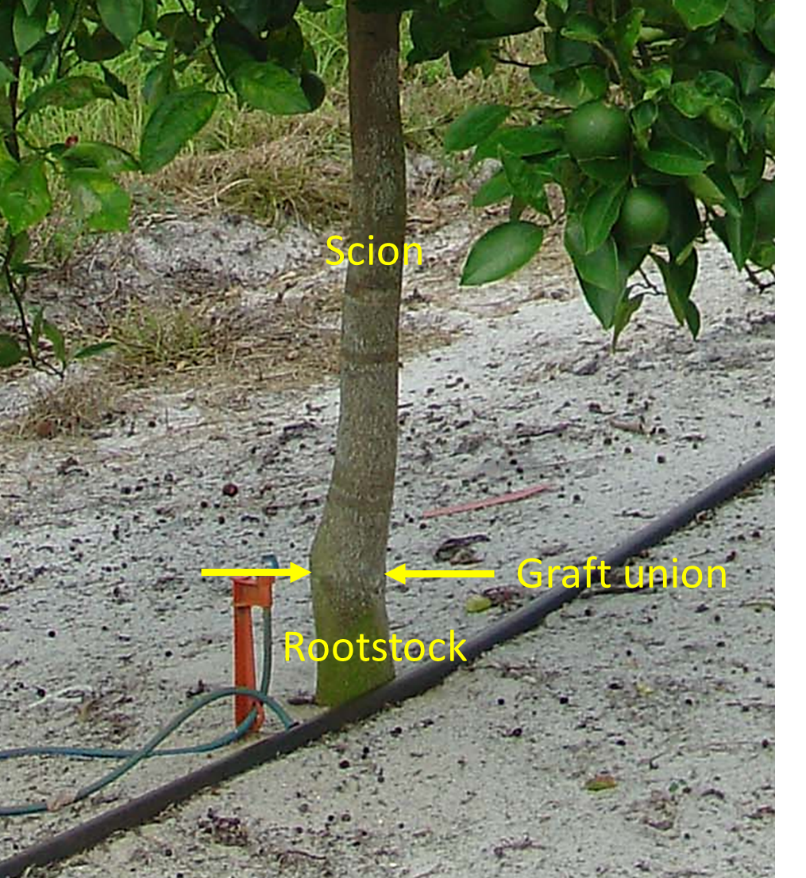


**Fig. S1.** Schematic diagram of the field study illustrating the experimental design (A); an untreated control plot (left) and a compost-treated plot (right) – trees are arranged in two rows on raised beds separated by furrows for drainage (B); a grafted citrus tree composed of scion and rootstock that are united at the graft union (C).

**
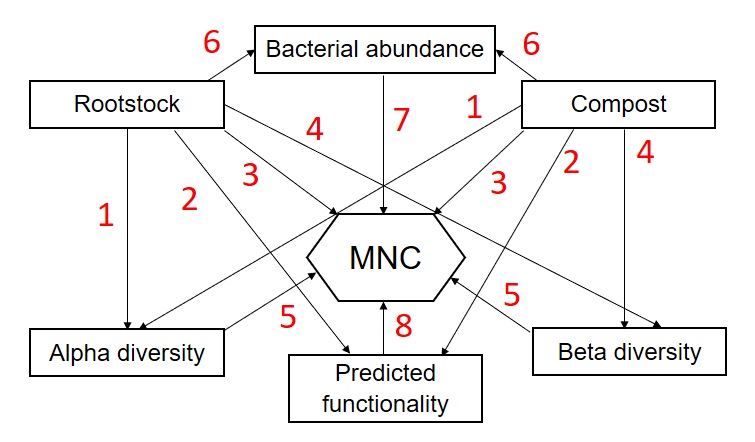
**

**Fig. S2.** A priori generic structural equation model (SEM) used in this study. The numbers in the arrows denote example references used to support our predictions (see References section)





**Fig. S3.** Root nutrient content of citrus trees on four different rootstocks. Soils were untreated (control) or treated with compost. Different letters above the bars indicate significant differences between rootstocks and treatments (linear mixed-effect model and Tukey's HSD; n = 8; *, *p* ≤ 0.05; **, *p* ≤ 0.01; ***, *p* ≤ 0.001). Values are expressed as mean with standard error


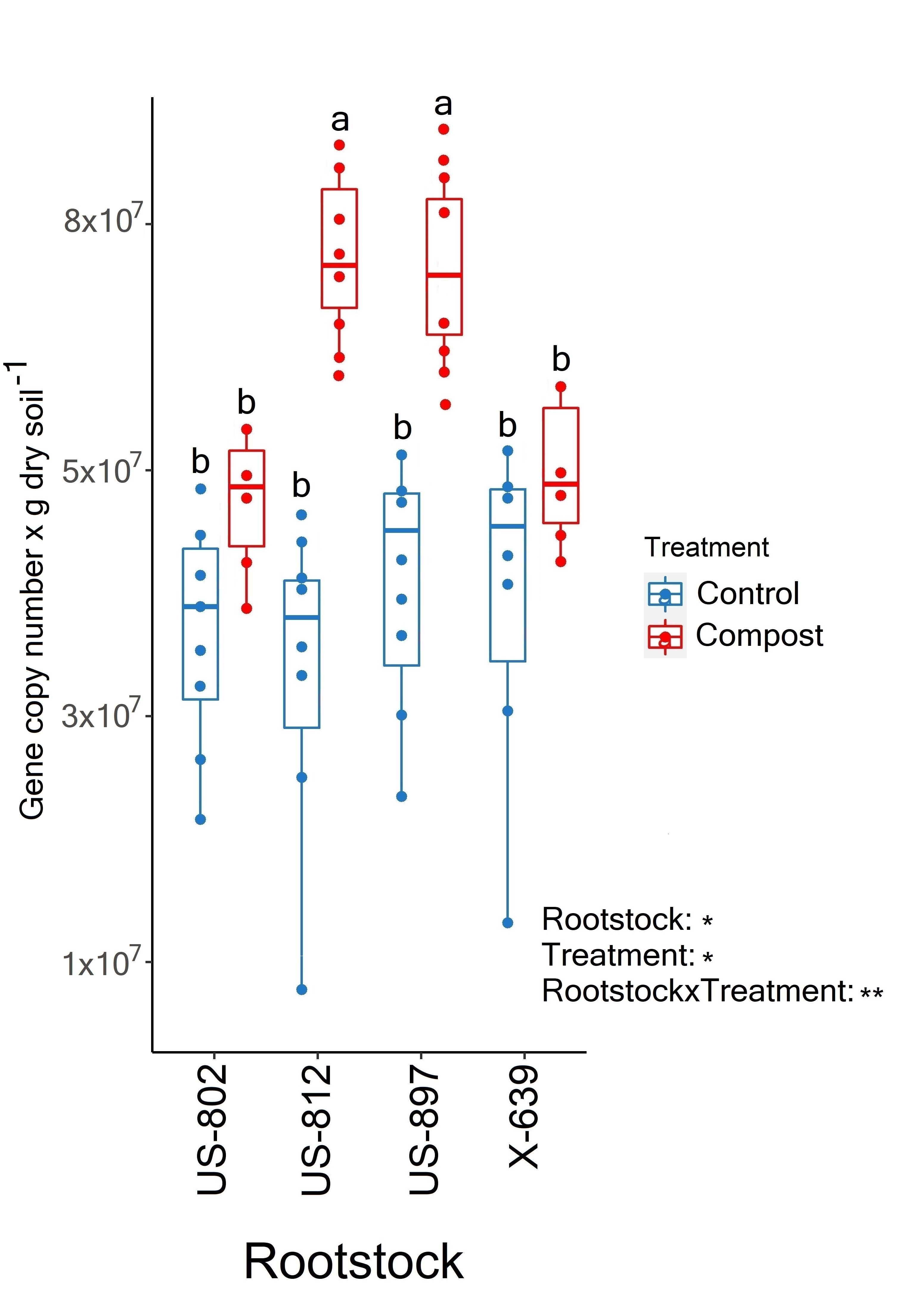


**Fig. S4.** Total abundance of active bacterial communities in the rhizosphere of citrus trees on four different rootstocks. Soils were untreated (control) or treated with compost. Different letters above the bars indicate significant differences between rootstocks and treatments (linear mixed-effect model and Tukey's HSD, n = 8; *, *p* ≤ 0.05; **, *p* ≤ 0.01; ***, *p* ≤ 0.001). Values are expressed as mean with standard error


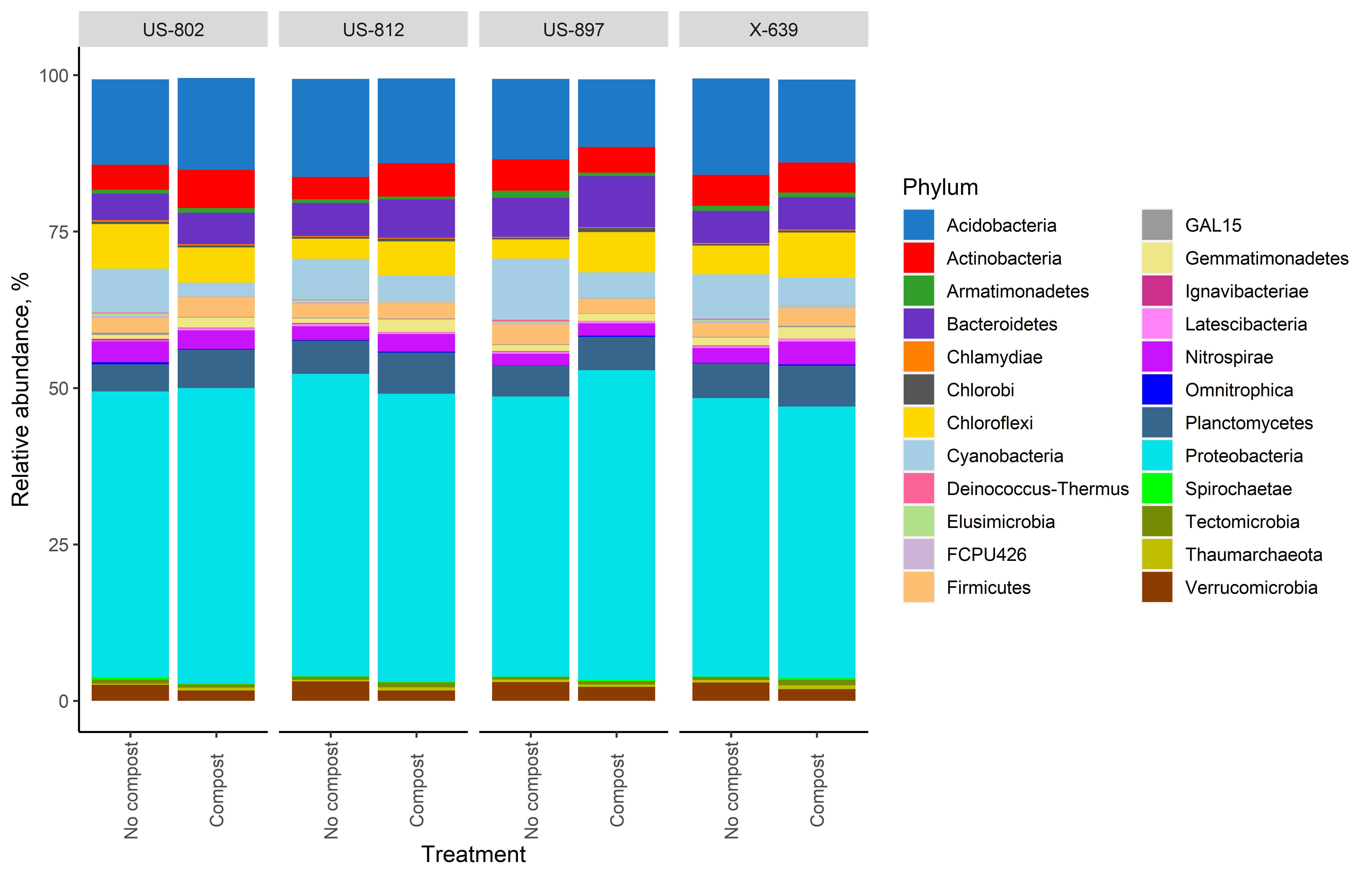


**Fig. S5.** Relative abundance of bacterial ASVs at the phylum taxonomic level in the rhizosphere of four different rootstocks. Soils were untreated (control) or treated with compost


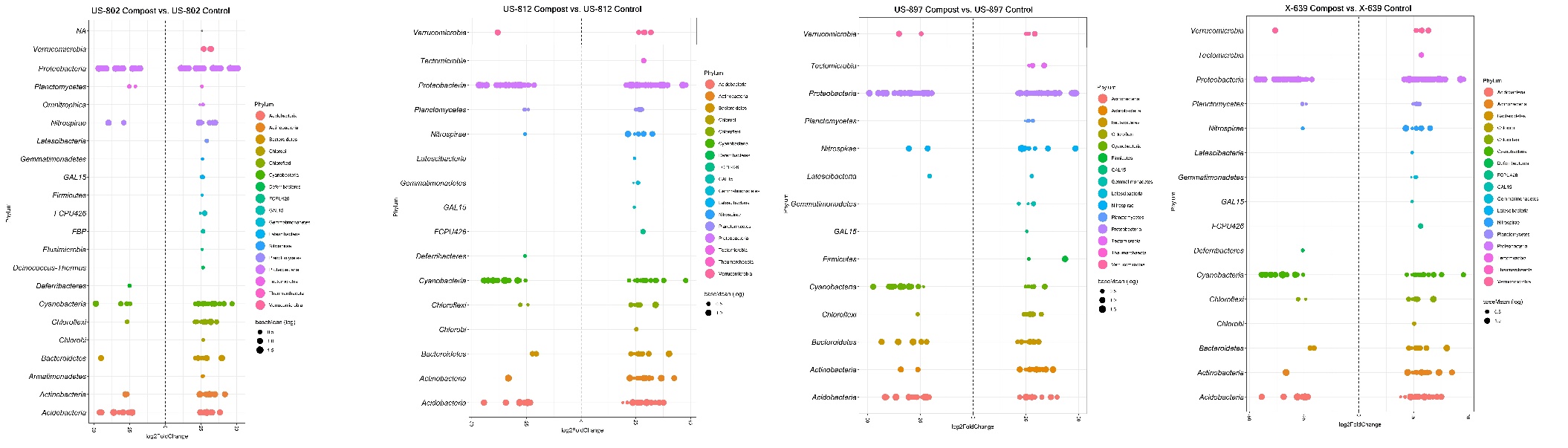


**Fig. S6.** Differentially abundant ASVs at the genus taxonomic level between compost and control treatments for each rootstock. The fold change is shown on the X axis and genera are listed on the Y axis. Each colored dot represents an ASV that was identified by DESeq2 analysis as significantly differentially abundant (*p* ≤ 0.05)


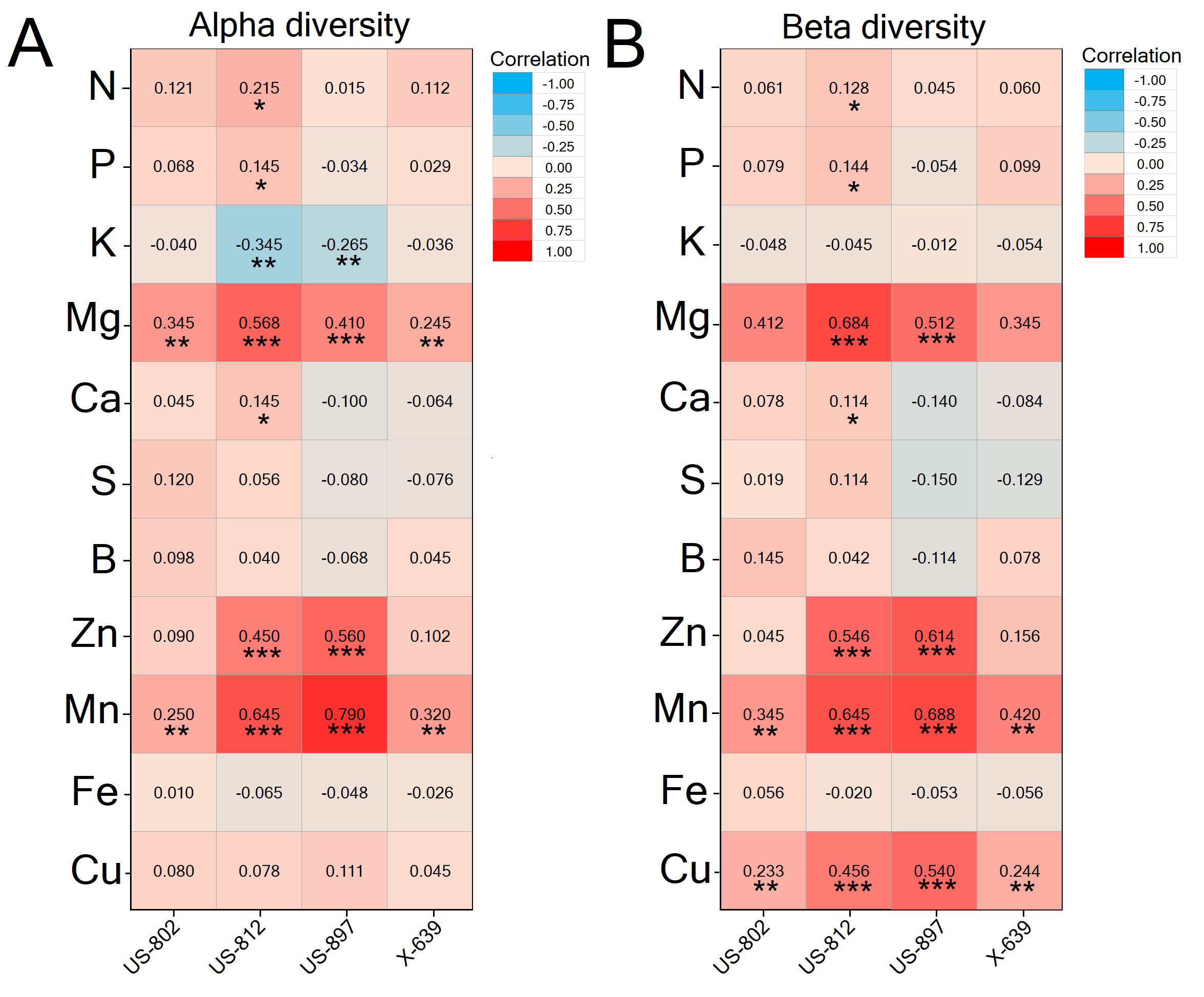


**Fig. S7.** Heatmaps of Spearman correlation coefficients between bacterial alpha (A) and beta (B) diversity and root nutrients for each rootstock. The shading from blue to red represents low-to-high positive correlation. *, *p* ≤ 0.05; **, *p* ≤ 0.01; ***, *p* ≤ 0.001

**
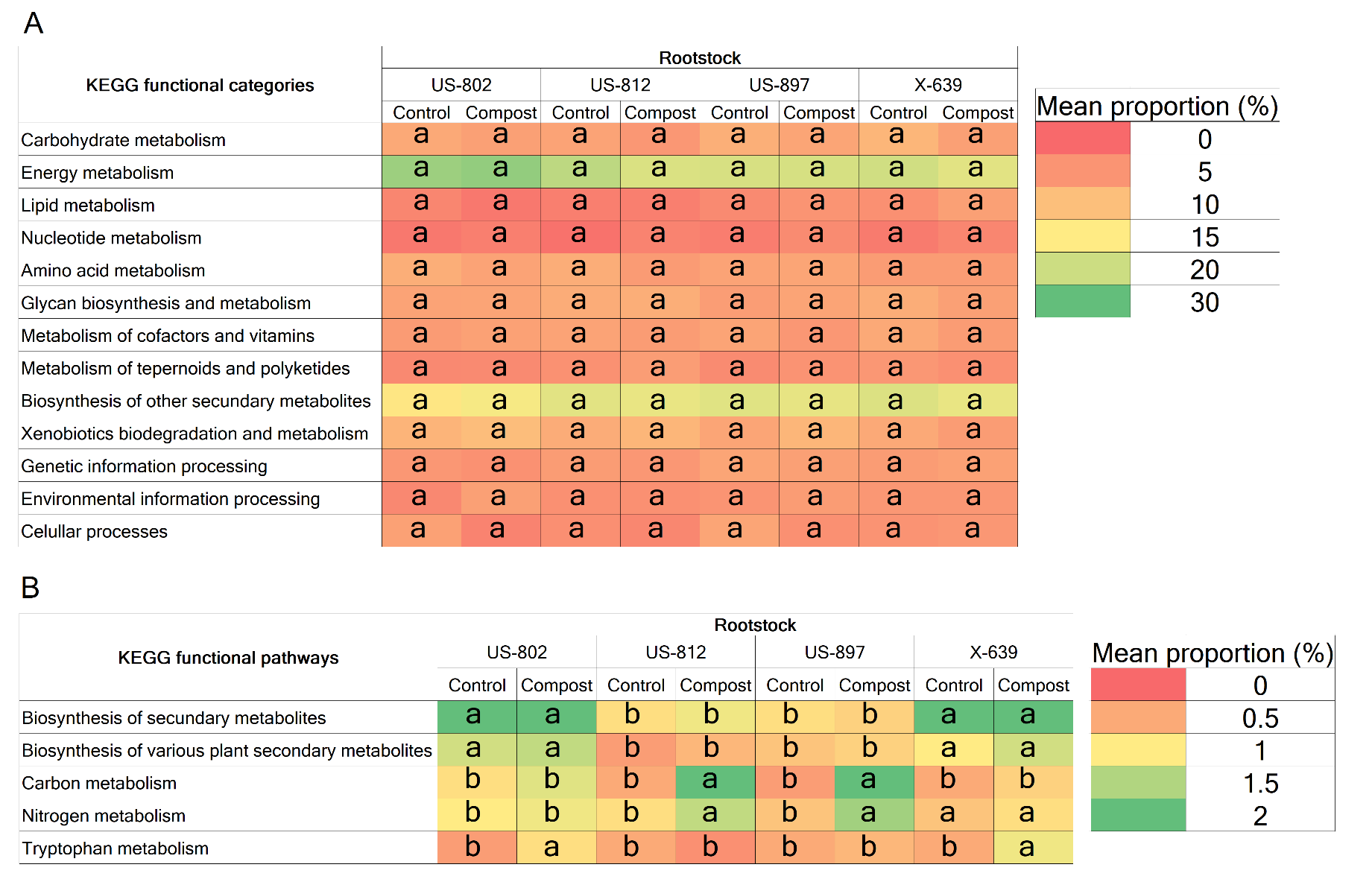
**

**Fig. S8.** Mean proportion of predicted KEGG categories (A) and pathways (B) in the rhizosphere of citrus trees on four different rootstocks. Soils were untreated (control) or treated with compost. For each row, different letters indicate significant differences between treatments and rootstocks (Tukey's HSD, *p* < 0.05; n = 8)

**References**

1,4. Castellano-Hinojosa A, Meyering B, Nuzzo A, Strauss SL, Albrecht U. Effect of plant biostimulants on root and plant health and the rhizosphere microbiome of citrus trees in huanglongbing-endemic conditions. Trees - Struct Funct. 2021;35:1525–39.

1, 2, 3, 4, and 6. Sharaf H, Thompson AA, Williams MA, Peck GM. Compost applications increase bacterial community diversity in the apple rhizosphere. Soil Sci Soc Am J. 2021;85:1105–21.

5, 7, and 8. D´Amico F, Candela M, Turroni S, Biagi E, Brigidi P, Bega A, et al. The rootstock regulates microbiome diversity in root and rhizosphere compartments of *Vitis vinifera* cultivar lambrusco. Front Microbiol. 2018;9:2240.

5, 7, and 8. Delgado-Baquerizo M, Maestre FT, Reich PB, Jeffries TC, Gaitan JJ, Encinar D, et al. Microbial diversity drives multifunctionality in terrestrial ecosystems. Nat Commun. 2015;7:1–8.

5, 7, and 8. Jiao S, Xu Y, Zhang J, Hao X, Lu Y. Core microbiota in agricultural soils and their potential associations with nutrient cycling. mSystems. 2019;4:e00313-18.

5, 7, and 8. Jiao S, Peng Z, Qi J, Gao J, Wei G. Linking Bacterial-fungal relationships to microbial diversity and soil nutrient cycling. mSystems. 2021;6:e01052-20.
